# Supplementary material for: Versatile non-luminescent color palette based on guest exchange dynamics in paramagnetic cavitands
Source: Nat Commun. 2021 May 24;12:3072. doi: 10.1038/s41467-021-23179-9 (PMC8144181; doi:10.1038/s41467-021-23179-9)
Supplement: Supplementary file 1 — Supplementary Information [file 41467_2021_23179_MOESM1_ESM.pdf]

Supplementary Information

Versatile Non-luminescent Colors based on Guest Exchange Dynamics in Paramagnetic Cavitands

Elad Goren[1], Liat Avram[2], Amnon Bar-Shir[1*]

[1] Department of Molecular Chemistry and Materials Science, Faculty of Chemistry, Weizmann Institute of Science, Rehovot, 7610001, Israel; [2] Department of Chemical Research Support, Faculty of Chemistry; Weizmann Institute of Science, Rehovot, 7610001, Israel

*Corresponding Author: amnon.barshir@weizmann.ac.il

SUPPLEMENTARY METHODS

Abbreviations

CD – Cyclodextrin, DTPA - Diethylenetriaminepentaacetic acid, DTPAA - Diethylenetriaminepentaacetic acid dianhydride, TEA – Triethylamine, DMSO – Dimethyl Sulfoxide, $LnCl_3 \cdot XH_2O$ – Lanthanide Chloride hydrate (X = 6/7, depends on the lanthanide), ddW - doubly distilled water.

Chemicals

$6^A,6^D$-diamino-$6^A,6^D$-dideoxy-α-cyclodextrin (**Diamino-CD**) was purchased from Arcos Organics; DTPAA and DMSO anhydrous were purchased from Sigma Aldrich. 4-Fluorobenzylamine (**1**) and 2,6-Difluorobenzylamine (**4**) were purchased from Apollo Scientific. 4-(Trifluoromethyl)-benzylamine (**2**) and 3,5-Difluorobenzylamine (**3**) were purchased from Alpha Aesar. Anthranilic acid (**5**) was generously obtained from the lab of Dr. Sergey N. Semenov. Praseodymium (III) chloride heptahydrate, Europium (III) chloride hexahydrate, Terbium (III) chloride hydrate, Dysprosium (III) chloride anhydrous, Holmium (III) chloride hexahydrate, Erbium (III) chloride hydrate, Thulium (III) chloride hydrate and Ytterbium (III) chloride hexahydrate were purchased from Strem Chemicals INC. Neodymium (III) chloride hydrate and Samarium (III) chloride hydrate were purchased from Alfa Aesar. Cerium (III) chloride heptahydrate and Lanthanum(III) chloride heptahydrate were generously obtained from the lab of Prof. Boris Rybtchinski.

α-Cyclodextrin-DTPA (CD-DTPA) Synthesis

Under N_2, a solution of $6^A,6^D$-diamino-$6^A,6^D$-dideoxy-α-cyclodextrin (**Diamino-CD**, 100 mg, 95.80 μmol), DTPAA (29.65 mg, 95.80 μmol) and TEA (one drop) was stirred in anhydrous DMSO (2 mL) overnight. The solution was added dropwise into cold acetone (60 mL) while stirring, and the resultant white powder

was collected after centrifugation (4˚C, 3000 rpm, 15 min). The crude solid was dissolved in a minimal volume of water and purified through a reversed phase liquid chromatography. **CD-DTPA** was isolated as a white powder upon the removal of solvent (13.74 mg, 21.6%); ^{1}H-NMR (500.08 MHz, D_2O) δ 4.99 (d, 2H), 4.97 (d, 2H), 4.94 (d, 2H), 4.14-4.04 (dd, 4H), 4.02-3.99 (d, 2H); 3.95-3.85 (m, 10H), 3.85-3.80 (dt, 2H), 3.80-3.72 (m, 4H), 3.72-3.65 (m, 2H), 3.65-3.59 (m, 2H), 3.61-3.52 (m, 10H), 3.47-3.36 (m, 4H), 3.40-3.32 (m, 4H), 3.32-3.22 (dd, 2H), 3.22-3.09 (m, 4H); ^{13}C-NMR (125.74 MHz, D_2O) δ 173.07, 170.65, 170.10, 101.60, 101.51, 101.48, 83.90, 82.05, 81.01, 73.44, 73.39, 72.81, 72.13, 71.75, 71.62, 71.53, 60.68, 60.12, 56.78, 55.98, 54.57, 50.60, 40.82; HRMS: $C_{50}H_{81}O_{36}N_5$ calcd: *m/z* 1327.4713, found: 1328.4739 $[M+H]^+$, Δ -2.0 ppm, 1350.4534 $[M+Na]^+$, Δ -1.9 ppm, 1366.4233 $[M+K]^+$, Δ -4.8 ppm.

[Lanthanide]-α-Cyclodextrin-DTPA (Ln-CD) Synthesis

For each lanthanide, a solution of **CD-DTPA** (one equivalent) and $LnCl_3$•XH_2O (one equivalent) in 15 mL ddW was refluxed under stirring for one hour. **Ln-CD** (90-100%) was isolated as a white powder upon the removal of solvent; HRMS: For **Dy-CD** - C50H79O36N5Dy calcd: *m/z* 1489.3796, found 1489.3787 $[M+H]^+$, Δ -0.6 ppm; HRMS spectra for **Ln-CD** with other lanthanides are available in the Supporting Information File (S22-S40).

Reversed Phase High Pressure Liquid Chromatography (RP-HPLC)

Analytical RP-HPLC analysis was performed using an Agilent Technologies 1260 Infinity quaternary pump LC system, equipped with a diode-array detector, through a C_{18} column. Preparative RP-HPLC was carried out using an Agilent 218 purification system, equipped with an auto-sampler, a C_{18} column, an UV-Vis dual wavelength detector and a 440-LC fraction collector, operating under OpenLab ChemStation software. Elution phases were composed of 0.1% TFA in ddW (eluent A) and 90% acetonitrile, 0.1% TFA in ddW (eluent B).

High-Resolution Electrospray Ionization Mass Spectrometry (ESI-Q-Tof-MS)

Analyses were carried out on a Waters Xevo G2-XS QTof Mass Spectrometer (Manchester, UK) with an electrospray ionization (ESI) source operating in the positive mode. Solutions were directly infused at a flow rate of 10 μL/min. All spectra were acquired in a mass range of 50 – 2000 m/z. Mass errors of the analyzed spectra are not larger than 5.0 ppm. The analyses were performed using a capillary voltage of 3.00 kV, a cone gas flow of 25 L/hr, a source temperature set at 120˚C, and a cone voltage of 20V. The desolvation temperature was set at 250˚C and the desolvation gas (N_2) flow rate was set to 400 L/hr. All measurements were done using Leucine-Enkephalin (200 μg/uL, acetonitrile:H_2O containing 0.1% formic acid (1:1, v/v)) as a lockspray reference, at a flow rate of 10 uL/min to ensure mass accuracy and to follow resolution mode. Data acquisition and recording were done by Waters MassLynx v4.2 software.

High-Resolution Nuclear Magnetic Resonance (NMR)

a. *Sample Preparation*: Unless stated otherwise, all NMR experiments were performed on solutions containing **Ln-CD** and a selected guest dissolved in D_2O (or in H_2O with a D_2O insert). Solutions were prepared in a 1:100 host-guest molar ratio with final concentrations of 150-170 μM and 15-17 mM respectively. All solutions were filtered using a MILLEX®-GV 0.22 μm PVDF filter unit prior to use.

b. *Data Acquisition*: All NMR experiments were performed on an 11.75 T AVANCEIII-HDNMR spectrometer (Bruker, Germany) with the sample temperature stabilized at 298K or 283K (for deletion experiments, Figure S48). *1D ^{1}H-NMR* spectra (500.08 MHz) were acquired for all samples prior to the ^{19}F-NMR experiments. *1D ^{19}F-NMR* spectra (470.54 MHz) were acquired for all host-guest samples, followed by longitudinal (T_1) and transverse (T_2) relaxation time evaluations. For structure assignment, ^{13}C{^{1}H} NMR (125.74MHz, 298K) was measured with a spectral width of 250 ppm, acquired with 64K points, 7800 scans and a recycle delay of 6.5 seconds. The ^{13}C pulse was set to 70 degrees to improve the sensitivity of the quaternary carbons.

c. *Relaxation times*: Prior to all GEST experiments, in order to adjust the experimental parameters (saturation time and recovery time), both inversion recovery (IR, for evaluating T_1) and Car–Purcell–Meiboom–Gill (CPMG, for evaluating T_2) experiments were performed.

d. *^{1}H-^{1}H COSY* (500.08MHz, 298K) experiment was acquired with a matrix of 2K (F2) by 512 (F1), zero-filled to 4K (F2) by 1K (F1), covering a spectral width of 2000 Hz. The number of scans was 24 with a recycle delay of 2 seconds affording an experiment time of 9 hours.

e. *^{1}H{^{13}C} HSQC* (500.08MHz, 298K) experiment was acquired in the phase sensitive mode using Echo/Antiecho-TPPI gradient selection with decoupling during acquisition. The spectral width was 5000 Hz for ^{1}H and 20121 Hz for ^{13}C with a matrix of 4K (F2) by 1k (F1), zero-filled to 8K (F2) by 2K (F1). The number of scans was 8 with a recycle delay of 1.5 seconds - total experiment time was 4.5 hours. CNST2 ($J_{C\text{-}H}$) was set to 145 Hz.

f. *^{1}H-^{1}H ROESY*: Two-dimensional ROESY experiment was acquired in the phase-sensitive mode. The Spectrum (Figure 2b) consisted of a matrix of 2048 (F2) by 128 (F1), zero-filled to 256, covering a sweep width of 3150 Hz. The Spectrum was obtained using a spin-lock pulse of 200 ms and recorded at 298 K with 76 scans.

g. *^{19}F-GEST* experiments: Following a repetition time equals to three to five times the T_1 of the studied solution (as evaluated from IR experiments), a presaturation continuous wave (CW) radiofrequency (RF) pulse, with a duration of T_1, was applied prior to the 90° radiofrequency pulse. The saturation pulse strength (B_1) was set to 2.5μT, unless stated otherwise in the text or in the figure captions. In order to acquire the full z-spectrum, the frequency of the presaturation pulse was swept from $\Delta\omega$ = +50 ppm to $\Delta\omega$ = -50 ppm offset relative to the resonance frequency of the free guest (except for guest **4** - $\Delta\omega$ = +80 ppm to $\Delta\omega$ = -80 ppm). In addition, a ^{19}F-NMR spectrum where the RF pre-saturation pulse was applied at ω = -0.21 ppm ($\Delta\omega$ = 116.10 ppm, 62.17 ppm, 110.52 ppm and 117.07 ppm for guests **1**, **2**, **3** and **4** respectively), where a saturation transfer effect was not expected, was acquired as a reference spectrum.

h. *Data Processing*: *The z-spectrum* was blueprinted for each experiment by plotting the normalized intensity (S/S_0 or I/I_0) of the ^{19}F free guest signal, at each frequency offset ($\Delta\omega$ relative to the frequency of the free guest), as a function of this applied presaturation pulse offset.

Magnetic Resonance Imaging (MRI)

a. *Sample Preparation*: Unless stated otherwise, all MRI experiments were performed on solutions containing **Ln-CD** and the stated guest dissolved in ddW. Solutions were prepared and loaded to achieve a 1:100/1:75 host-guest molar ratio with final concentrations of 170 μM/227 μM and 17 mM respectively. All solutions were filtered using a MILLEX®-GV 0.22 μm PVDF filter unit prior to use. (i) *Words/PIN codes* encoding experiments (Figure 4) involve loading of different **Ln-CD** in the same well, for part of the wells, and make use of additional host-guest solutions in 1:25/1:33/1:50 ratios (guest concentration has kept the same). Each well was loaded with 25/33/50 μL solution, with the respective concentration, depending on the required number of lanthanides (4/3/2 respectively), to yield a final 1:100 host-guest concentration for each **Ln-CD** inside the well. *Barcodes* and *Code Manipulation* experiments (Figure 4 and 5 respectively) include the loading of 1:75 host-guest solutions on a 384-well plate, which was cut to a 3×3 well size prior to use (different **Ln-CD** in each well). Each well was filled with a volume of 100 μL host-guest solution. (iii) *Code Deletion* experiment (Figure 5) utilizes the loading of 70 μL 1:100 host-guest solutions at each well (different **Ln-CD** in each well), and then adding 50 μL of Anthranilic acid solution (**5**, 30.14 mM in ddW) to each well, in order to eliminate the paraGEST effects. (iv) *Concealed Code* experiments require the employment of a custom build apparatus, which is composed from three layers including 3×3 wells surfaces. Each well was loaded with 40 μL of 1:100 host-guest solution.

b. *Data Acquisition*: MRI experiments were performed on a 15.2 T horizontal scanner (BioSpec, Bruker), using a $^{1}H/^{19}F$ double-tuned radiofrequency (RF) volume coil with a 23 mm inner diameter. Bruker's ParaVision 6 (PV6) was used as the preclinical imaging software. ^{1}H images were obtained using a Rapid Acquisition with Refocused Echoes (RARE) sequence with a repetition time (TR) of 2000 msec, an echo time (TE) of 33.41 msec, a RARE factor = 8, a 20×20 cm^2 field of view (FOV), and a 256×256 size matrix. In addition, same images were acquired using a Localizer sequence for comparison - TR = 46.25 msec, TE = 1.46 msec, flip angle = 90°, FOV = 20×20 cm^2, and matrix size =256×256 size matrix. Images were converted to a DICOM format using PV6's image reconstruction module.

2D Experiments (Barcodes, Words, PIN codes, Code Manipulation and *Code Deletion*) – The working frequency for ^{19}F images was determined from the free guest's ^{19}F-frequency. ^{19}F single pulse sequence with TR = 6000 msec and a 90° excitation pulse was used to measure the SFO1 frequency. ^{19}F images were obtained using a RAREst sequence with TR = 6,000 msec, TE = 10.55 msec, RARE factor = 16, FOV = 20×20 cm^2 and matrix size = 32×32. Images were converted to a DICOM format using PV6's image reconstruction module. ^{19}F-GEST images were taken using a RAREst sequence, modified with a CEST module. The same parameters as for ^{19}F images were used in each experiment, with the addition of a 1.9 μT (*Barcodes, Code Manipulation* and *Code Deletion*) or a 2.5 μT (*Words* and *PIN codes*) saturation pulse (B_1) radiated for 2000 msec in different offsets.

c. *Concealed Code experiments* – SFO1 was measured for each layer separately using an Image-Selected In vivo Spectroscopy (ISIS) sequence with TR = 6,000 msec. ^{19}F images were collected using a RAREst sequence with TR = 6,000 msec, TE = 17.59 msec, RARE factor = 16, FOV = 50×50 cm^2 and matrix size = 64×64. Images were converted to a DICOM format using PV6's image reconstruction module.

^{19}F-GEST images were taken using a RAREst sequence, modified with a CEST module. The same parameters as for ^{19}F images were used in each experiment, with the addition of a 2.5 μT saturation pulse (B_1) radiated for 2000 msec in different offsets.

d. *Data Processing*: *z-spectra* were designed for each region of interest (ROI, i.e., each well) using a custom-built Matlab code, based on the principles stated above. The saturation transfer effect on the free guest, i.e., the MTR or GEST effect, was calculated for each voxel in the image by using a Lorentzian line shape fitting, as previously described.[1, 2] MTR maps at specific offsets were constructed through ImageJ 1.52a, by subtracting selected images (i.e., images at offsets where saturation is observed) from images taken at their symmetrical chemical offsets (i.e., negative $\Delta\omega$ from the same size positive $\Delta\omega$ and the opposite), or at edge offsets (i.e., the largest positive/negative $\Delta\omega$), utilized as M_0 offsets. Images were filtered using median or mean (average) ImageJ filters to improve SNR. The resultant ^{19}F-GEST images were adjusted to a 256×256 matrix size and assigned with the designated colors by changing their lookup tables (LUTs). MTR maps for the middle layer in the *Concealed Code experiment* were constructed through MatLab from a fitted GEST data. The GEST data was fitted according to data collected from a Water Saturation Shift Referencing (WASSR)[3] sequence, acquired for the same slice geometry in the range of $\Delta\omega$ = -2 ppm to $\Delta\omega$ = 2 ppm with B_1 = 1.9 μT.

SUPPLEMENTARY DISCUSSION

NMR Assignment

While native CDs exhibit high symmetry, which allows a relatively easy characterization by NMR, partial modifications on their glucose units can leads to the splitting of the spin systems due to symmetry breaks. This results in a crowded spectrum with an overly-splitted, overlapped, broad aliphatic area, as evident only from Diamino-CD's NMR spectra (Supplementary Figures 1 and 2).

CD-DTPA exhibits three spin-systems (Supplementary Figures 4-9), which can be mainly evident by the location of three representing peaks for the anomeric H-1 protons (i.e., H-1, H-1' and H-1'', Supplementary Figure 5, 4.94-4.98 ppm), indicating three types of CD's sugar units. To complete the characterization, advanced 2D-NMR experiments, such as COSY, HSQC and ROESY (Supplementary Figures 10-19), were performed. 2D COSY NMR (Supplementary Figures 10-13) had allowed us to first identify the CD-DTPA's inner protons (H-1 to H-6) and assign their "ranges". Following that, 2D HSQC NMR (Supplementary Figures 14-17) had enabled to distinguish between most of CD-DTPA's inner protons/carbons (H/C-1 to H/C-5) to the DTPA-bridge's protons/carbons (H/C-7 to H/C-11) and H-6, based only on the correlations' phases (i.e., blue and green, representing secondary and tertiary carbons respectively). Moreover, this experiment indicates the existence of inequivalent protons on several carbons (C-6, C-8 and C-9, indicated by "a" and "b"). The two H-6 protons undergo dramatic changes in their chemical shifts due to the DTPA modification. 2D ROESY NMR (Supplementary Figures 18 and 19) had enabled the identification of an H-4 peak underlying H-2's multiplet, due to a "through-space" interaction between H-4 and H-1.

Considering the intricate NMR spectra and broadside chemical shifts (summarized at Supplementary Tables 1 and 2), characterization was mainly based on MS analysis (Supplementary Figures 20-40).

SUPPLEMENTARY FIGURES

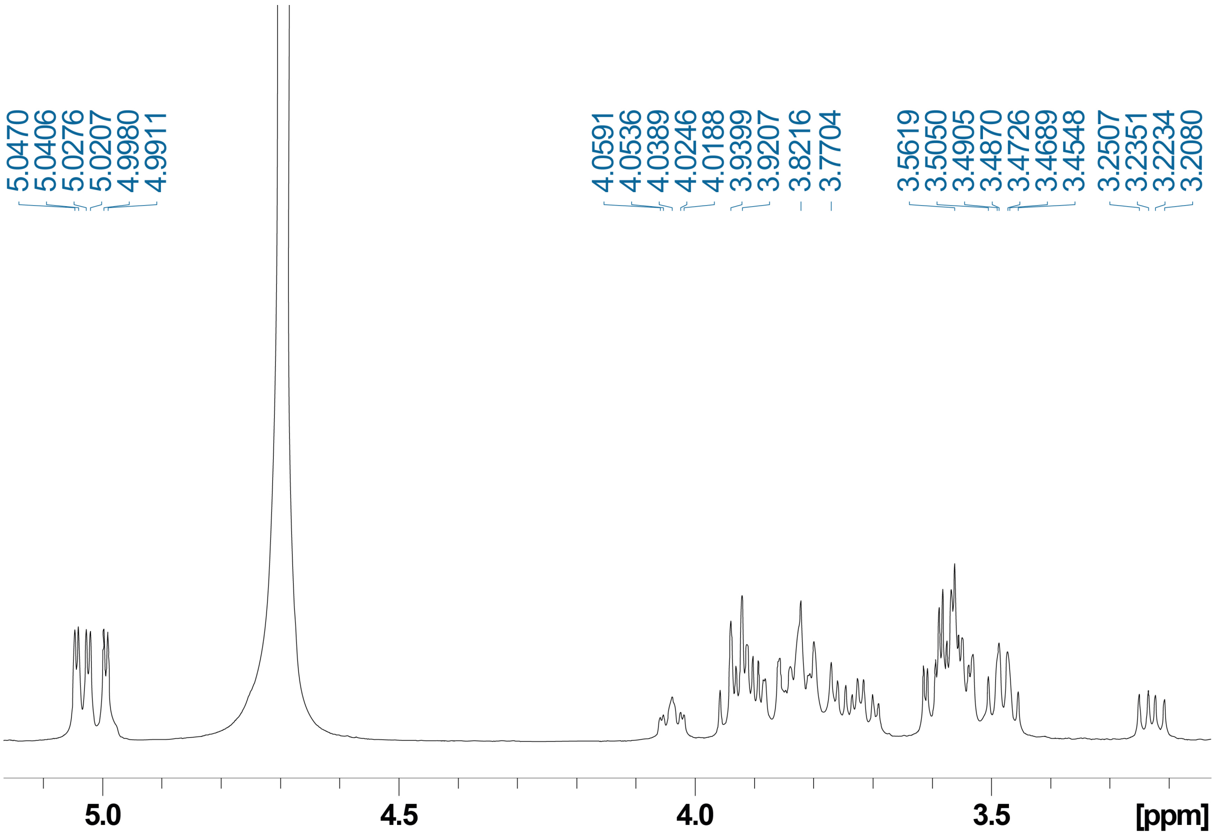

Supplementary Figure 1. ^{1}H-NMR spectrum (500.08 MHz, D_2O) for $6^A,6^D$-diamino-$6^A,6^D$-dideoxy-α-cyclodextrin (Diamino-CD).

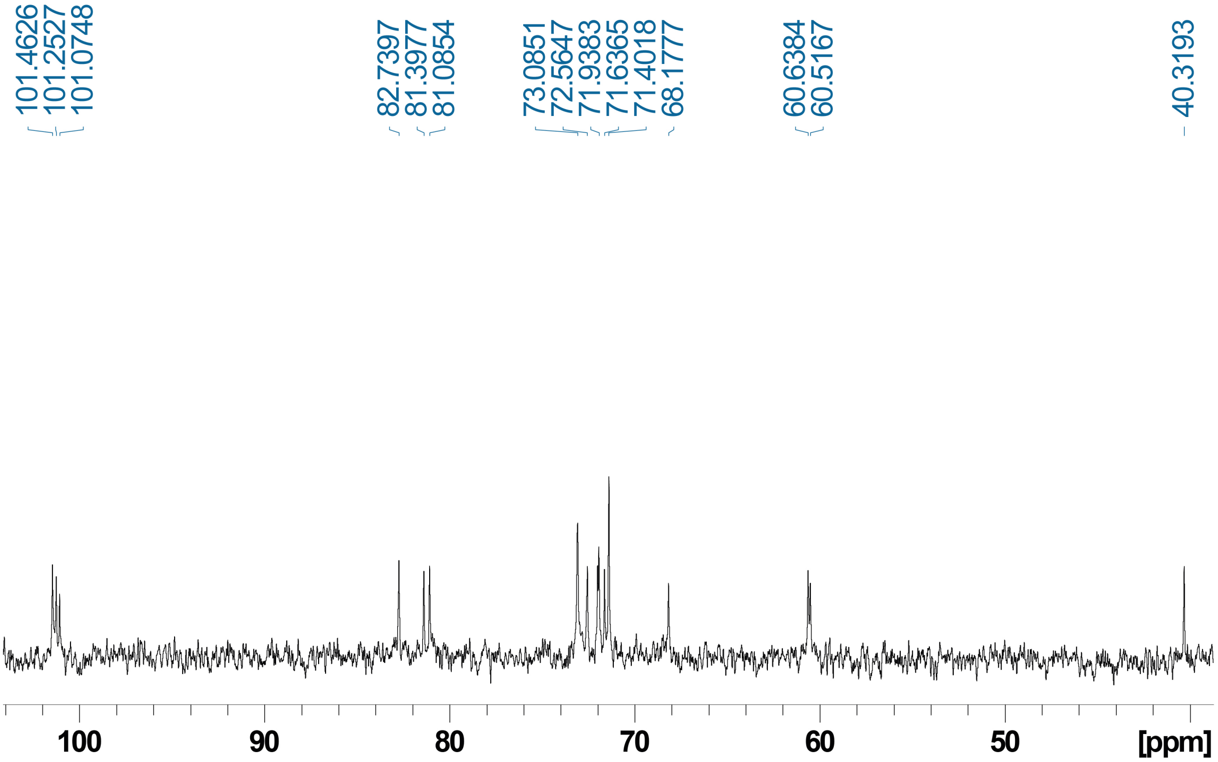

Supplementary Figure 2. $^{13}C\{^{1}H\}$ NMR spectrum (125.74 MHz, D_2O) for $6^A,6^D$-diamino-$6^A,6^D$-dideoxy-α-cyclodextrin (Diamino-CD).

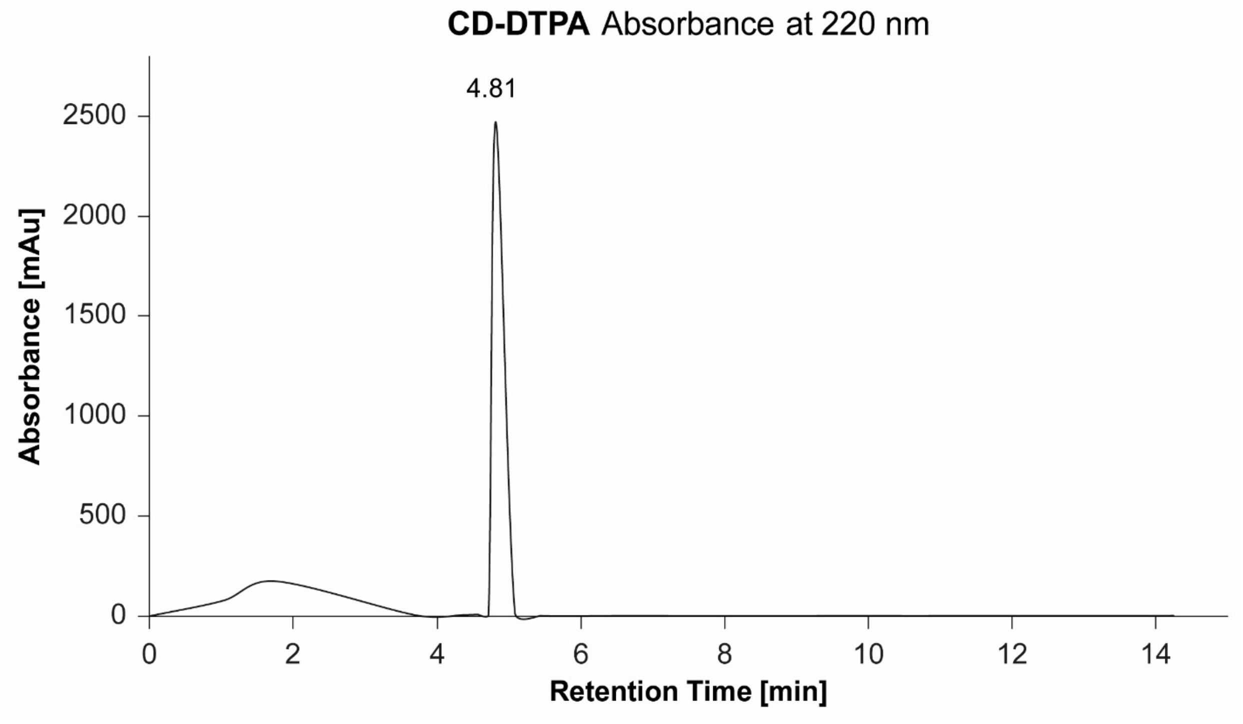

Supplementary Figure 3. Cyclodextrin diethylenetriaminepentaacetic acid (CD-DTPA) absorbance at 220 nm. Measured at analytical High Pressure Liquid Chromatography (HPLC).

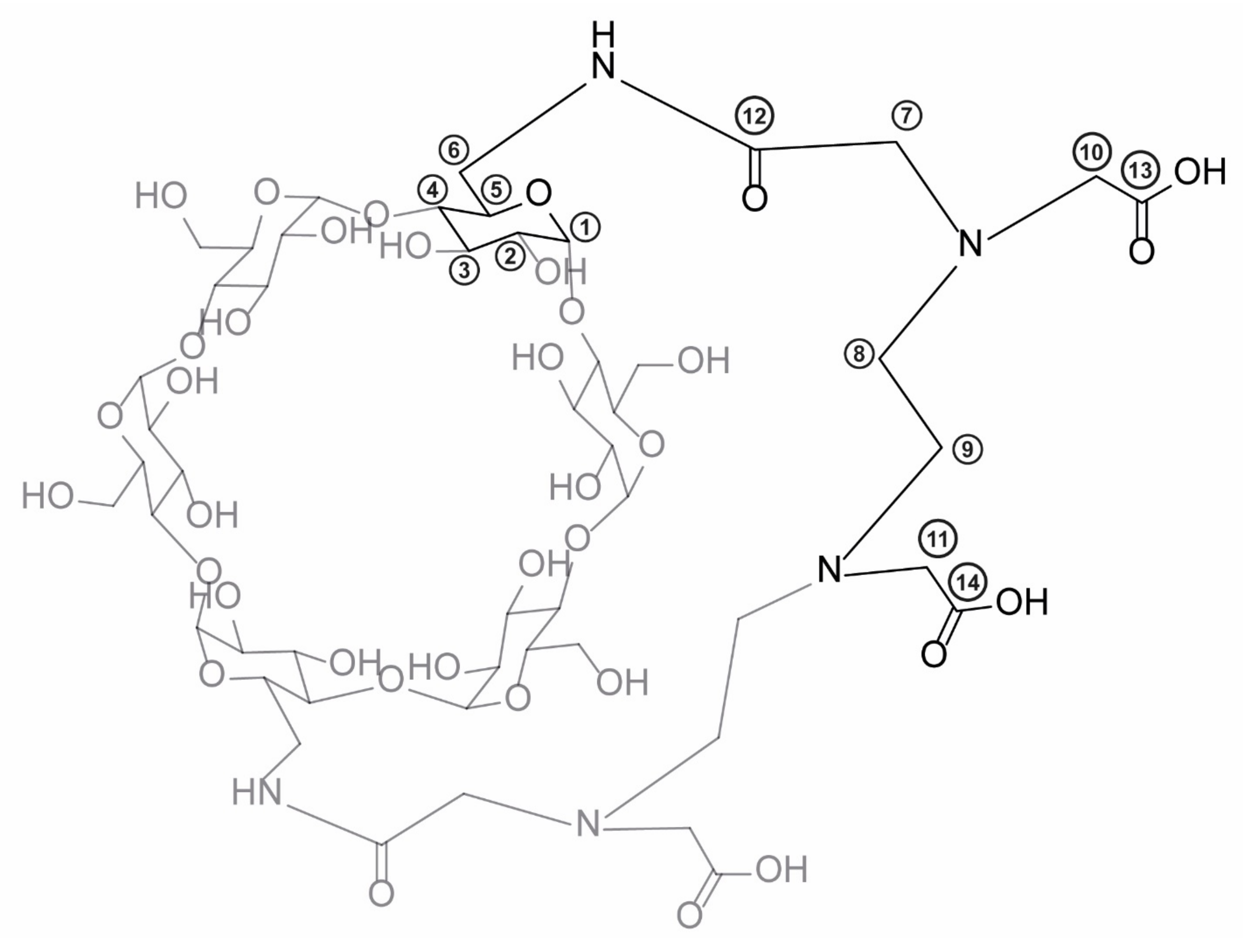

Supplementary Figure 4. Cyclodextrin diethylenetriaminepentaacetic acid (CD-DTPA) structure and atom indications.

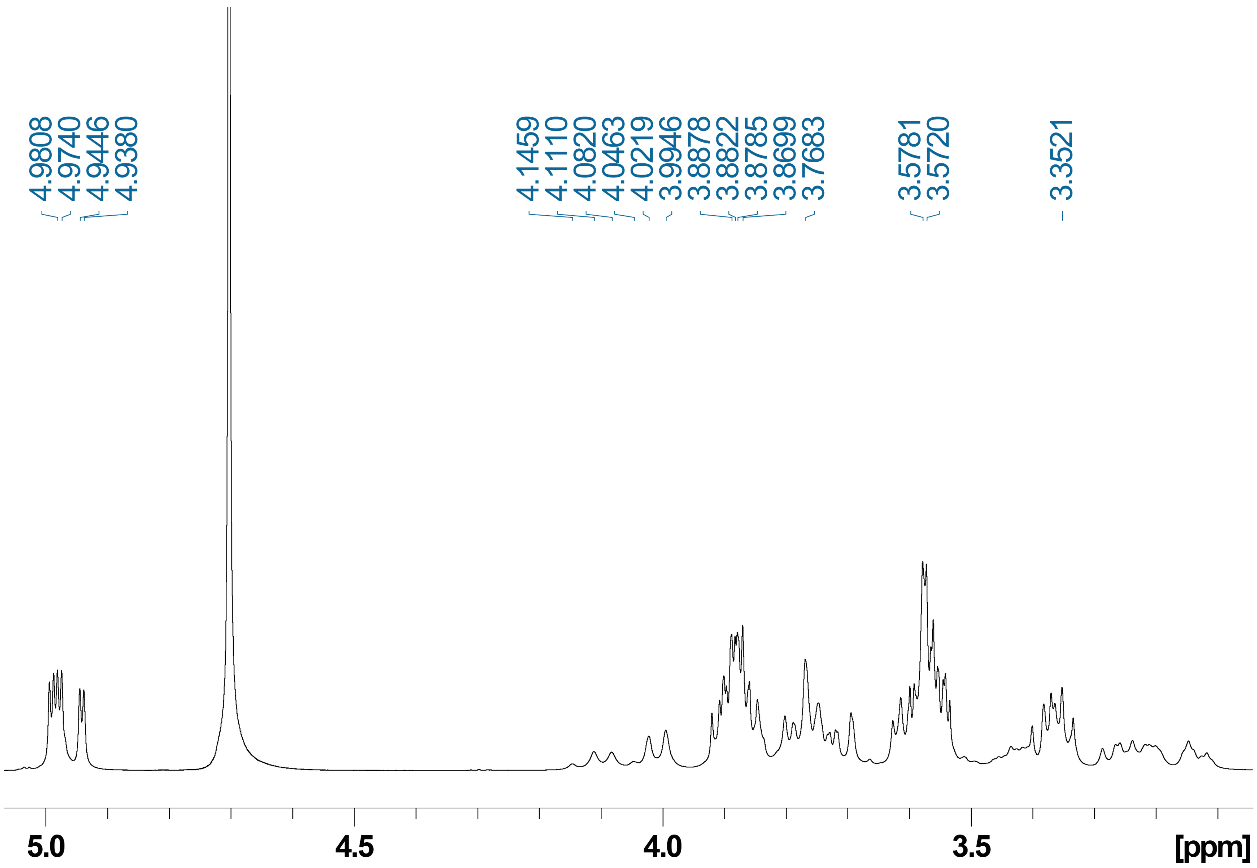

Supplementary Figure 5. **^{1}H-NMR spectrum (500.08 MHz, D_2O) for Cyclodextrin diethylenetriaminepentaacetic acid (CD-DTPA).**

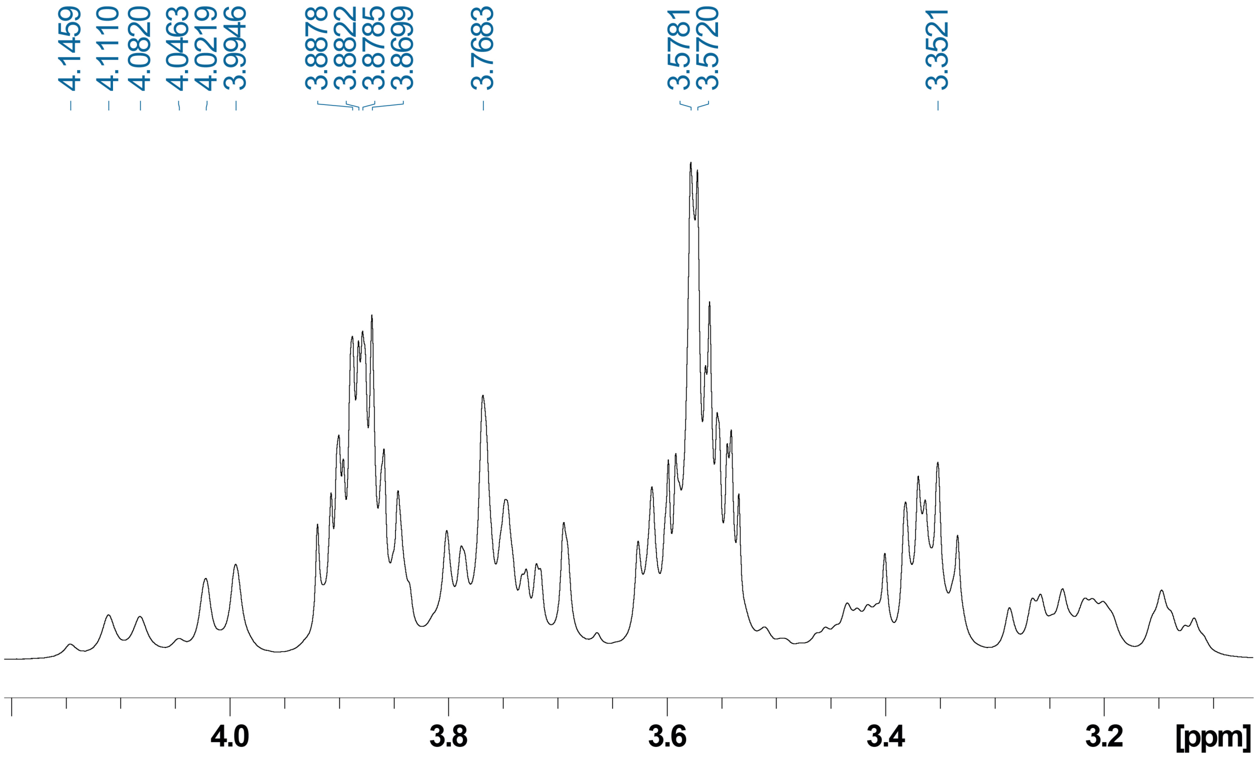

Supplementary Figure 6. Region of ^{1}H-NMR spectrum (Supplementary Figure 5) showing peaks for H-2 to H-11 of Cyclodextrin diethylenetriaminepentaacetic acid (**CD-DTPA**).

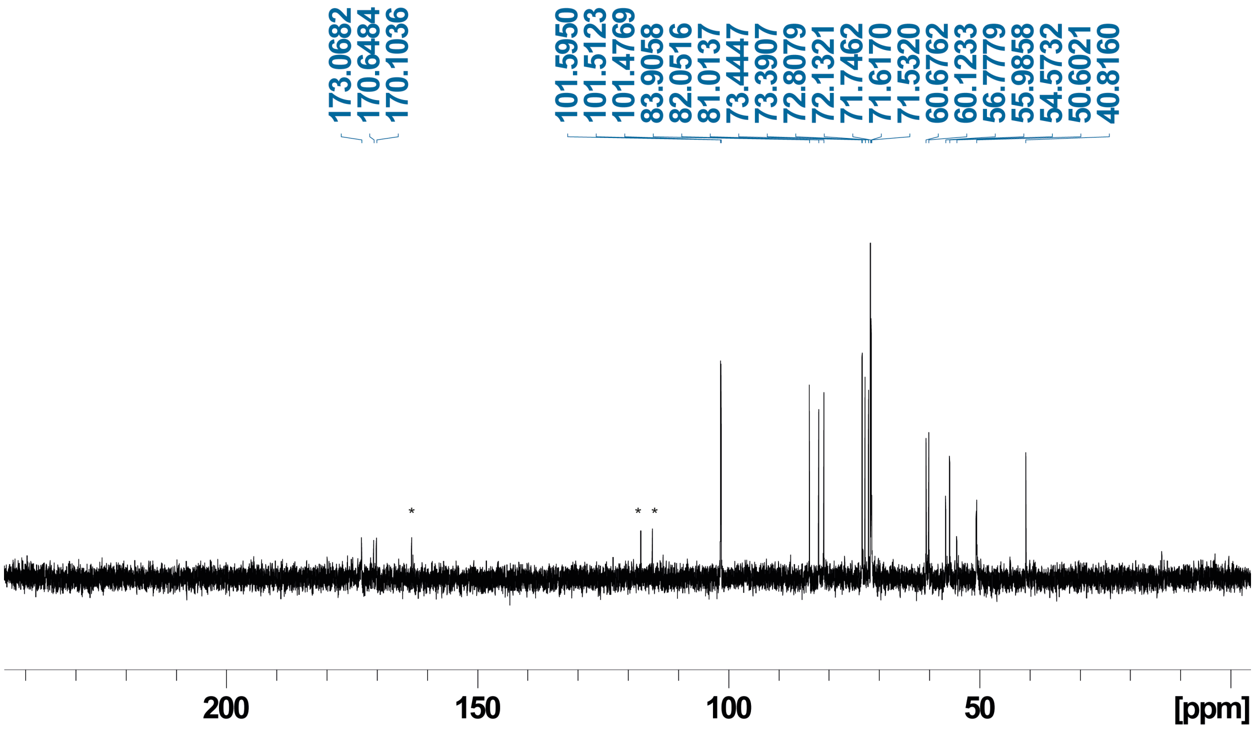

Supplementary Figure 7. $^{13}C\{^{1}H\}$ **NMR spectrum (125.74 MHz,** D_2O**) for Cyclodextrin diethylenetriaminepentaacetic acid (CD-DTPA)**. * Peaks representing TFA contaminations.

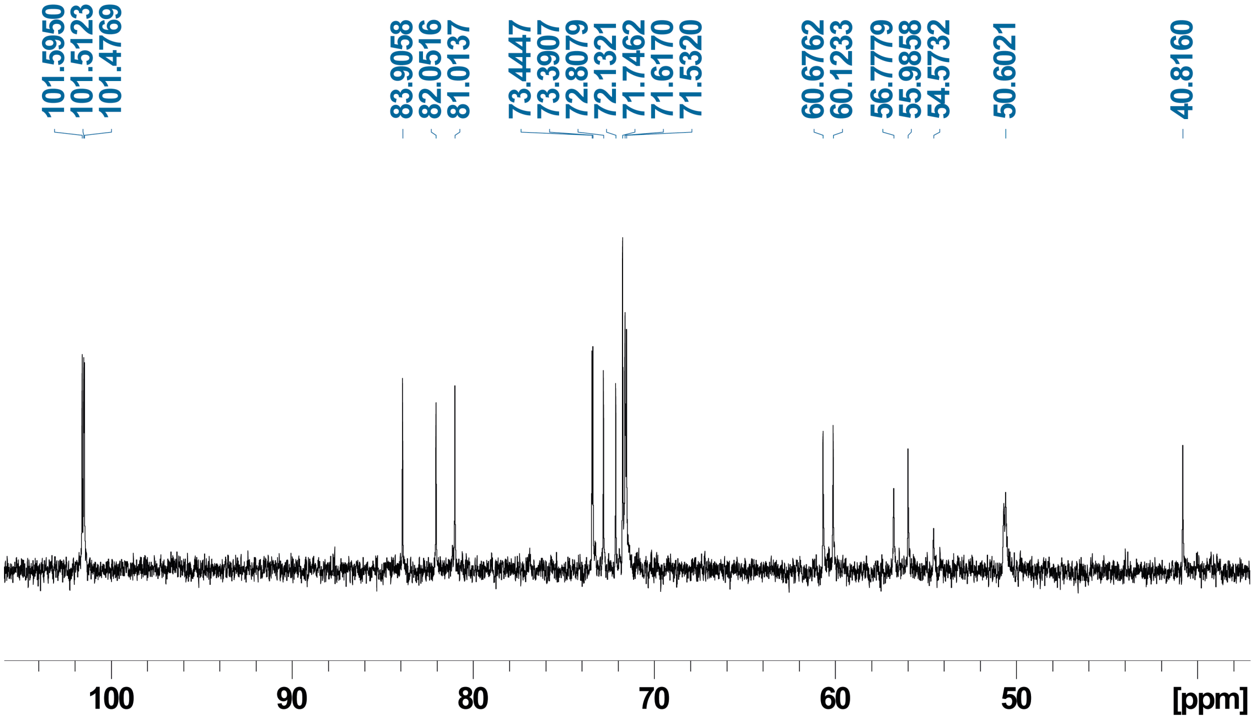

Supplementary Figure 8. Region of $^{13}C\{^{1}H\}$ NMR spectrum (Supplementary Figure 7) showing peaks of C-1 to C-11.

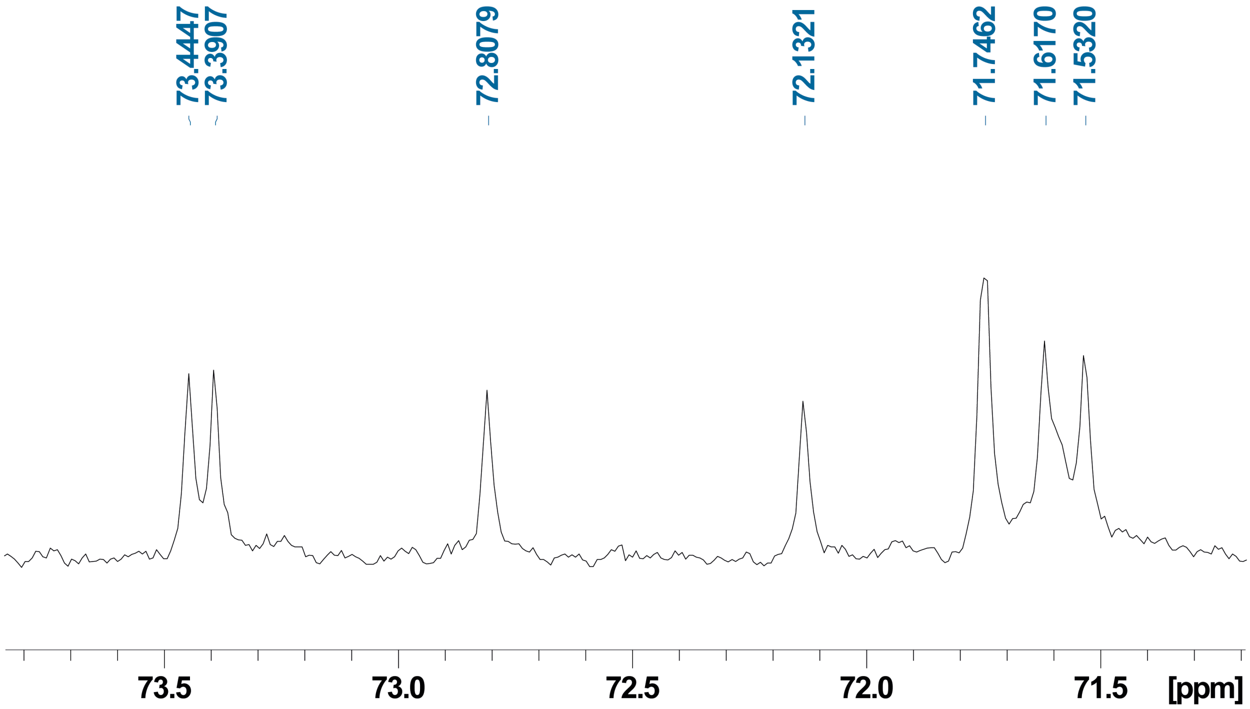

Supplementary Figure 9. Part of $^{13}C\{^{1}H\}$-NMR spectrum (Supplementary Figure 7) showing peaks for three types of C-2, C-3 and C-5.

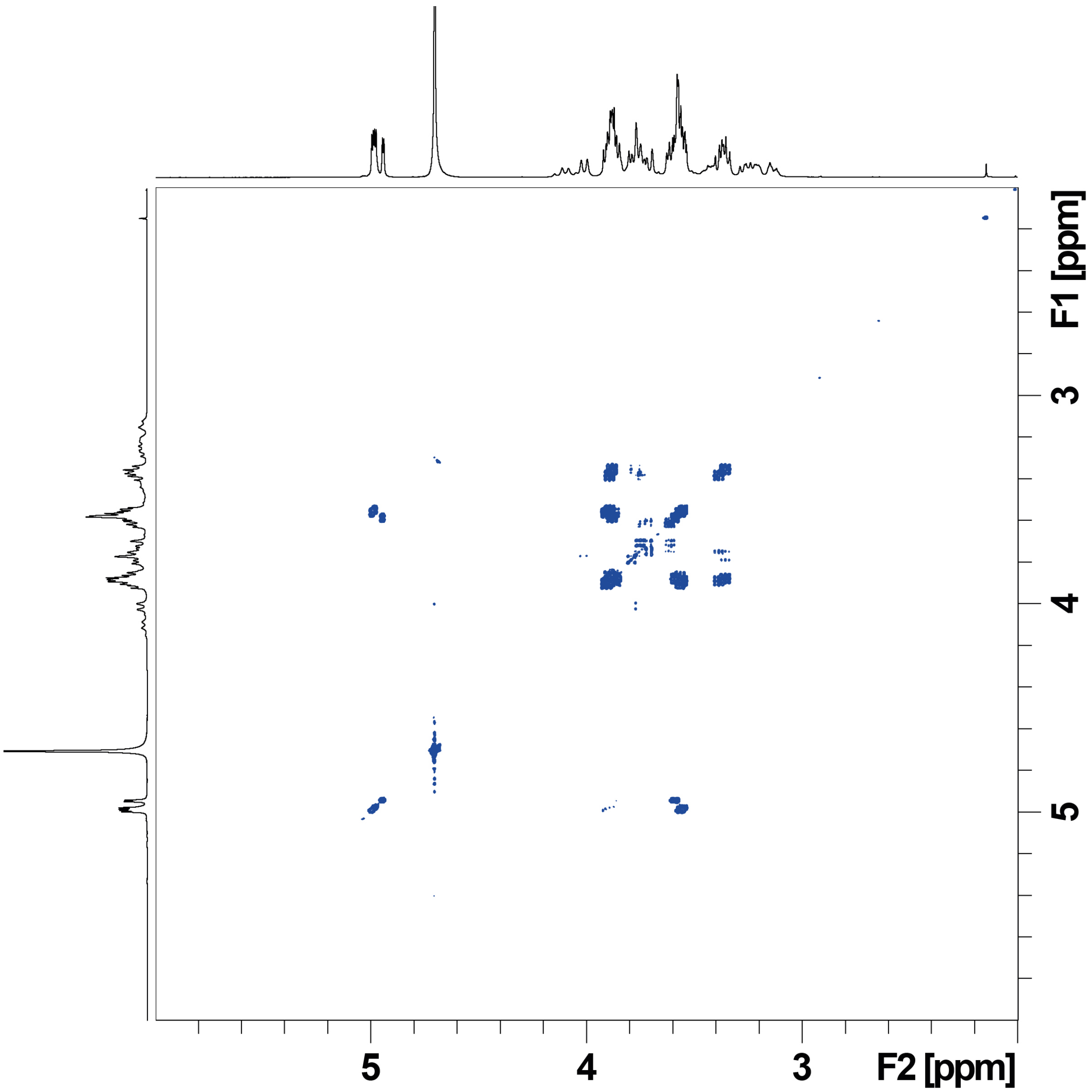

Supplementary Figure 10. ^1H-^1H COSY spectrum (500.08 MHz, D_2O) for Cyclodextrin diethylenetriaminepentaacetic acid (CD-DTPA).

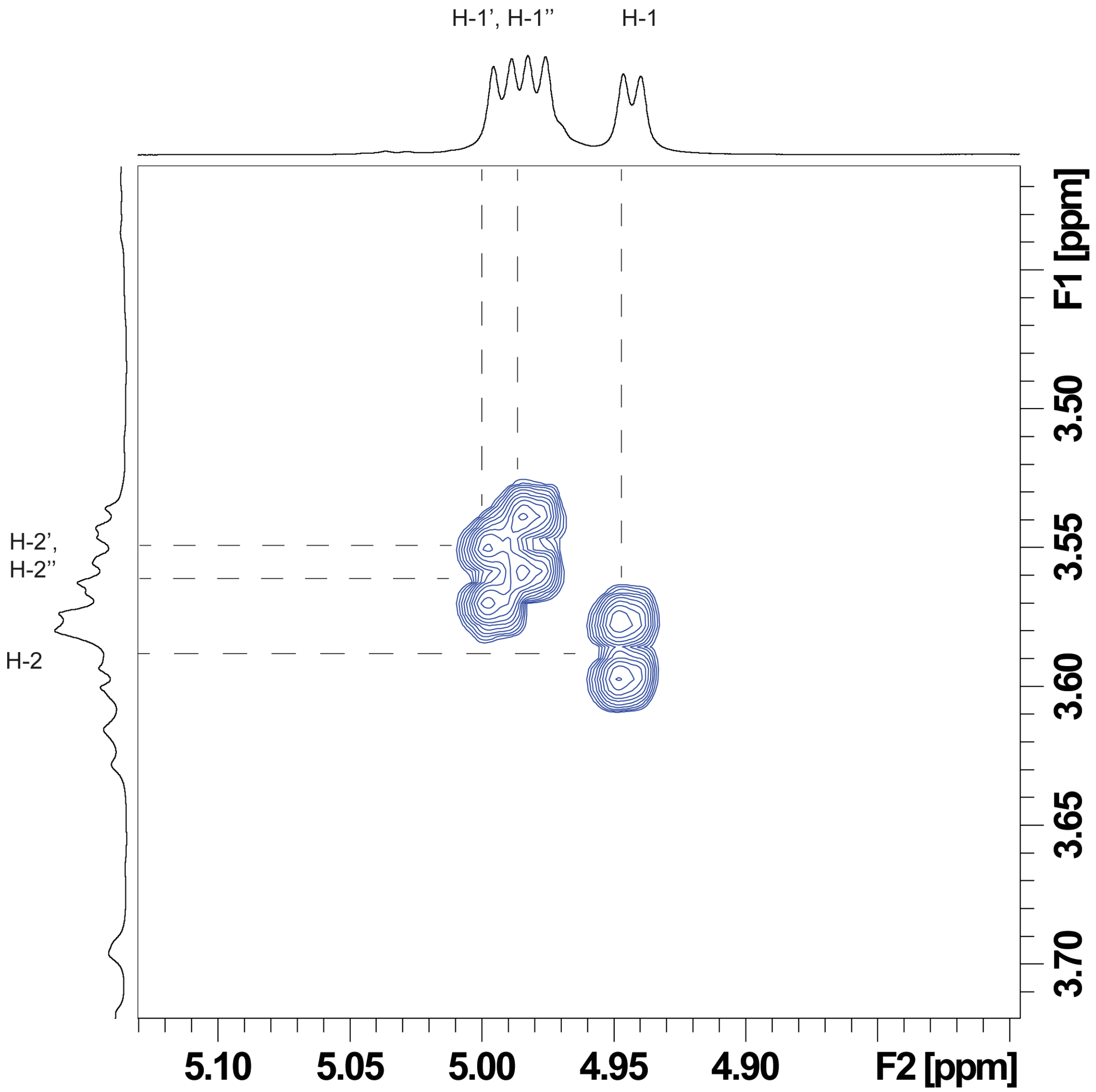

Supplementary Figure 11. Region of ^{1}H-^{1}H COSY spectrum (Supplementary Figure 10) showing the interactions between H-1, H-1', H-1'' (F2) and H-2, H-2', H-2'' (F1).

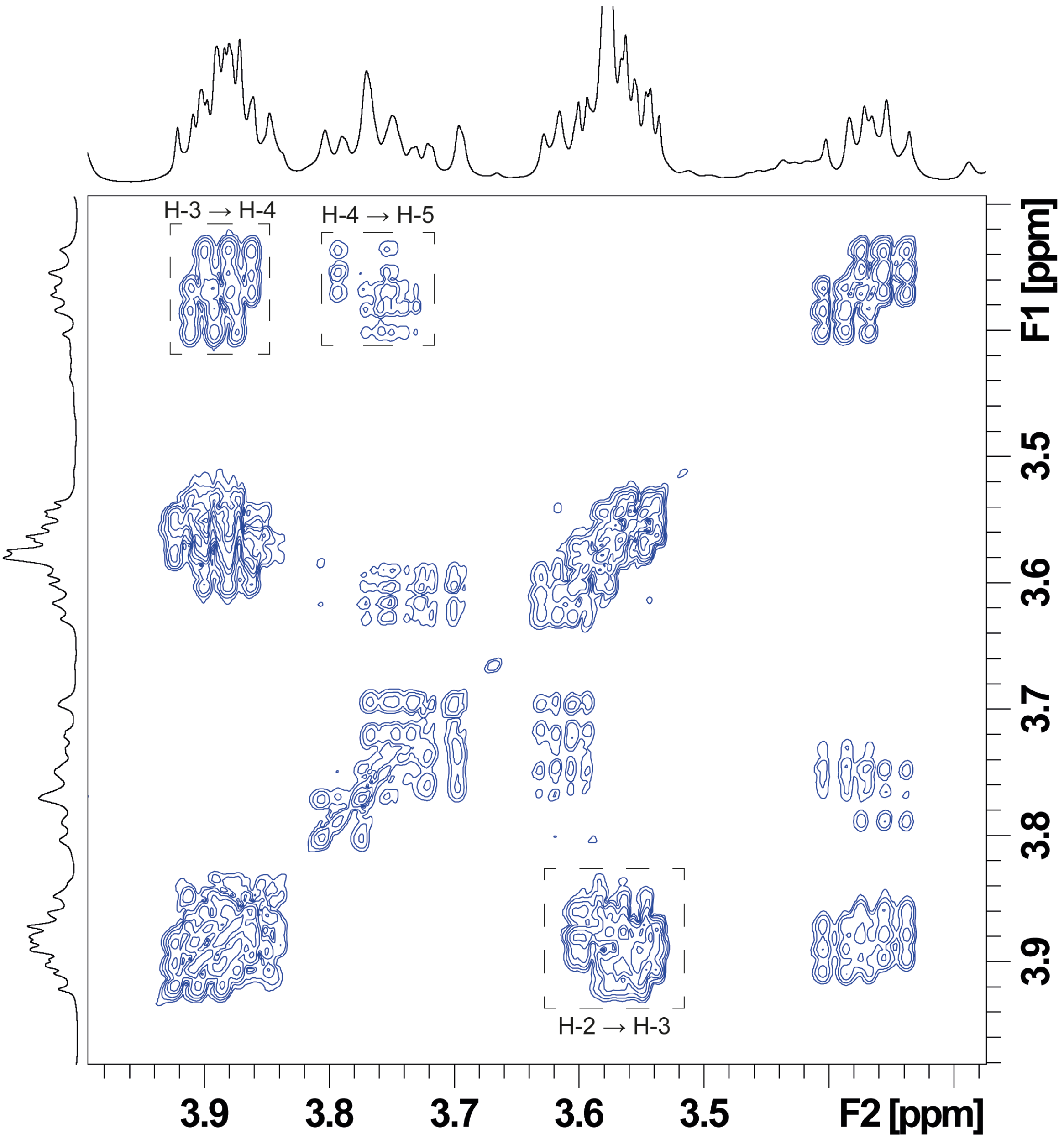

Supplementary Figure 12. Part of ^{1}H-^{1}H COSY spectrum (Supplementary Figure 10) showing interactions between **CD-DTPA**'s ring protons (H-2 to H-5).

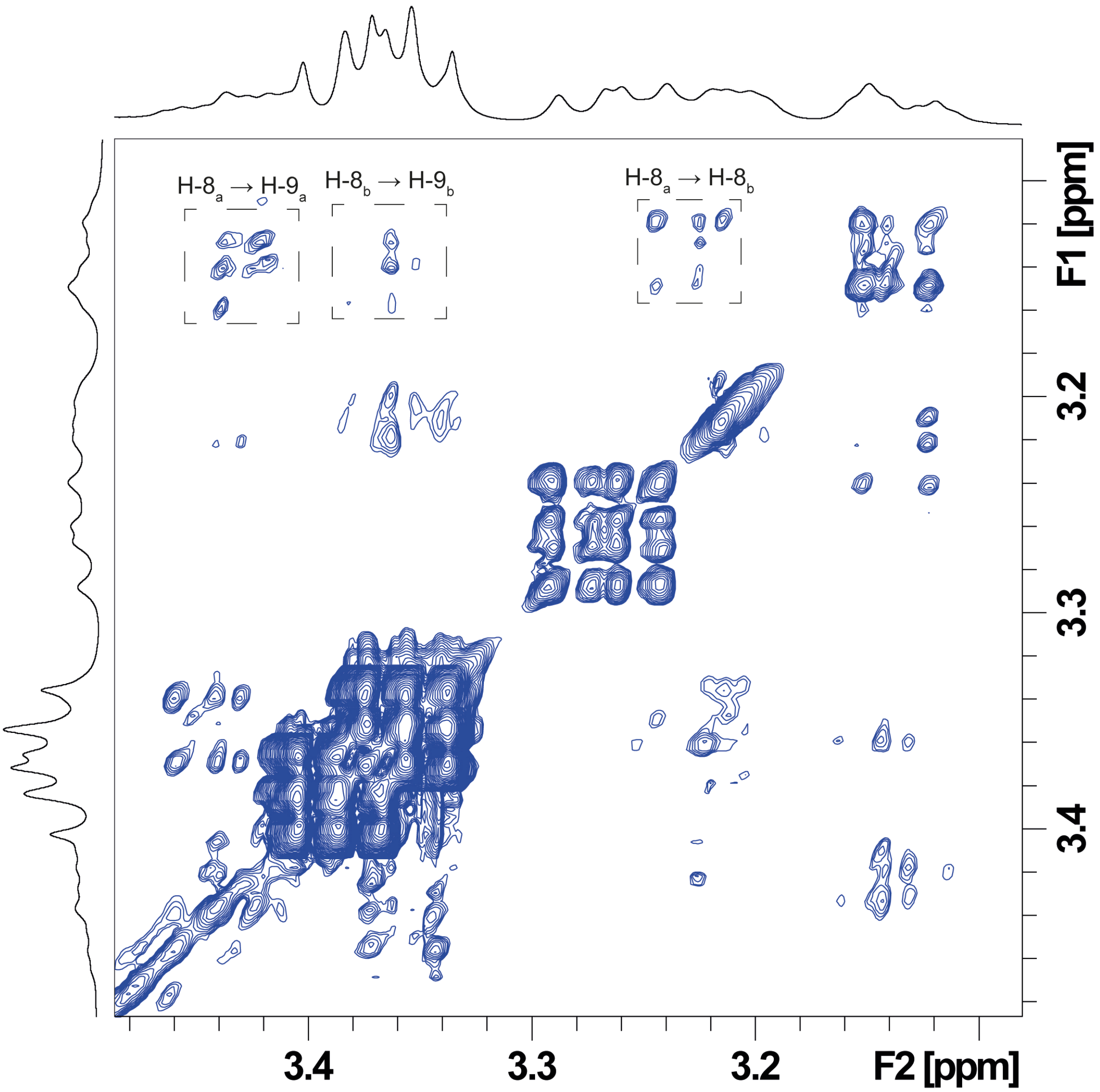

Supplementary Figure 13. Part of ^{1}H-^{1}H COSY spectrum (Supplementary Figure 10) showing interactions between **CD-DTPA**'s bridge protons H-$8_{a/b}$ and H-$9_{a/b}$.

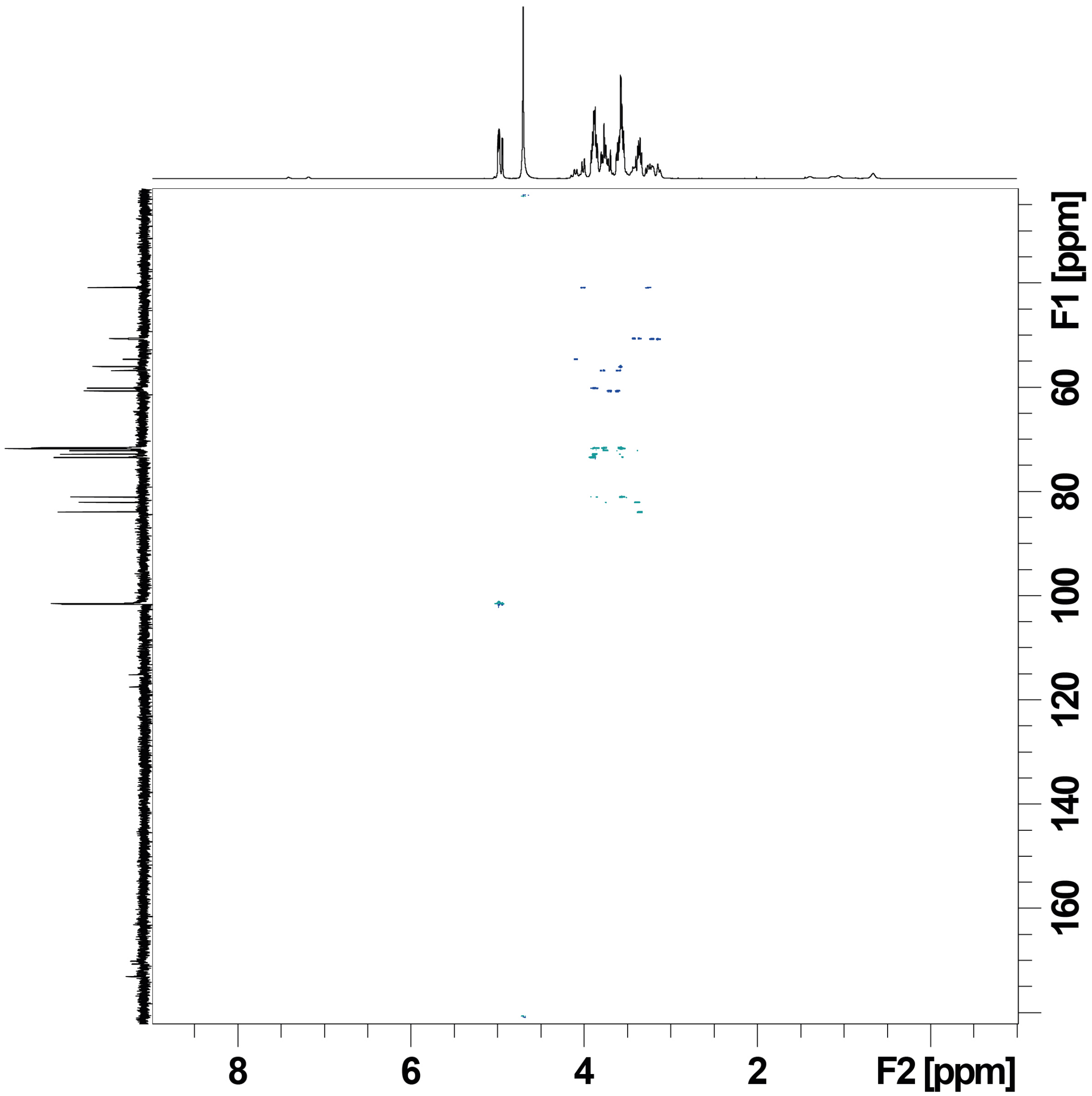

Supplementary Figure 14. ^1H-^{13}C HSQC spectrum (500.08 MHz, 125.74 MHz, D_2O) for Cyclodextrin diethylenetriaminepentaacetic acid (CD-DTPA).

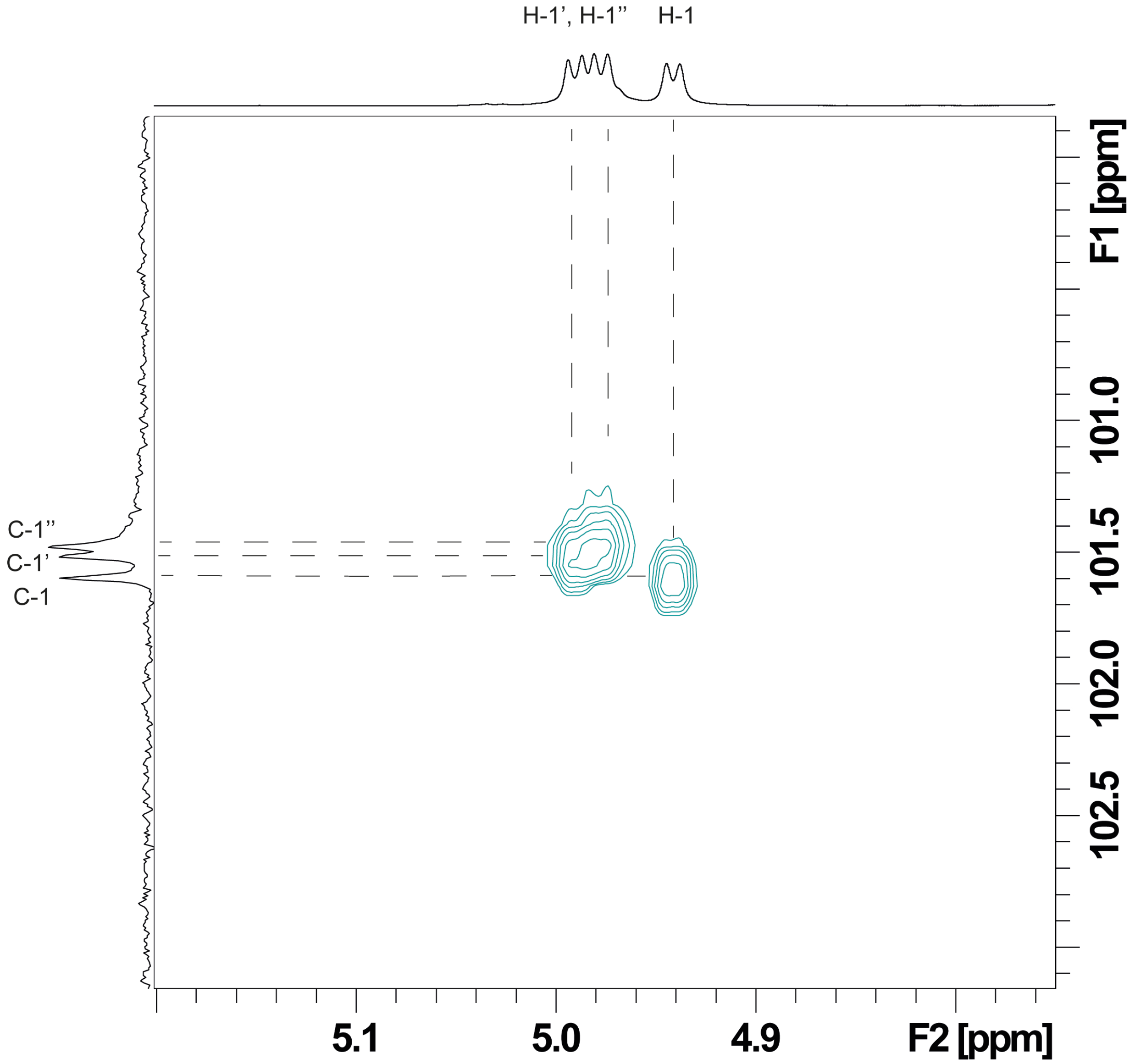

Supplementary Figure 15. Region of ^{1}H-^{13}C HSQC spectrum (Supplementary Figure 14) showing H/C-1 correlations.

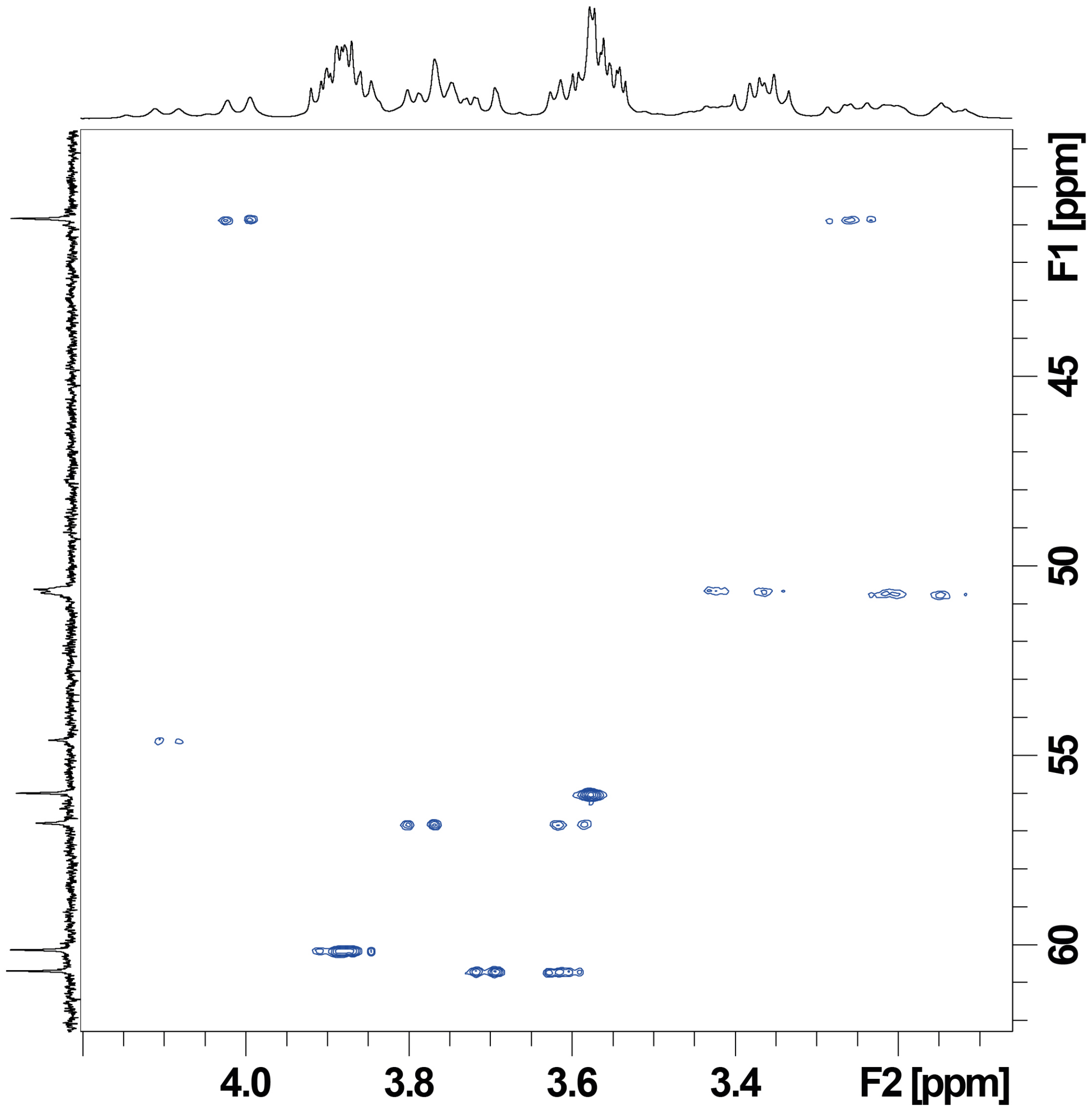

Supplementary Figure 16. Region of ^{1}H-^{13}C HSQC spectrum (Supplementary Figure 14) showing CH_2 correlations (H/C-6 to H/C-11).

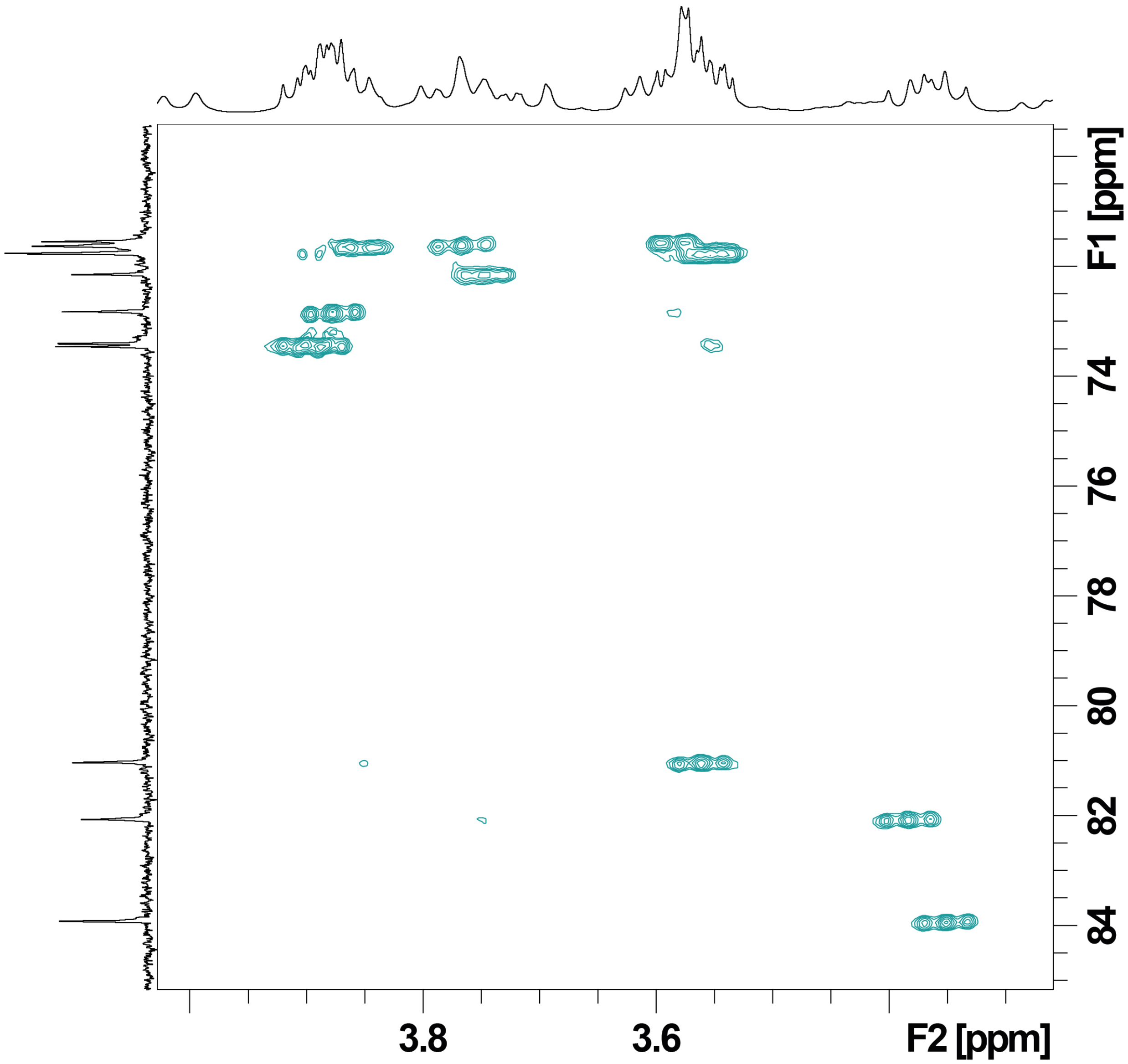

Supplementary Figure 17. Part of ^{1}H-^{13}C HSQC spectrum (Supplementary Figure 14) magnification showing CH correlations (H/C-2 to H/C-5).

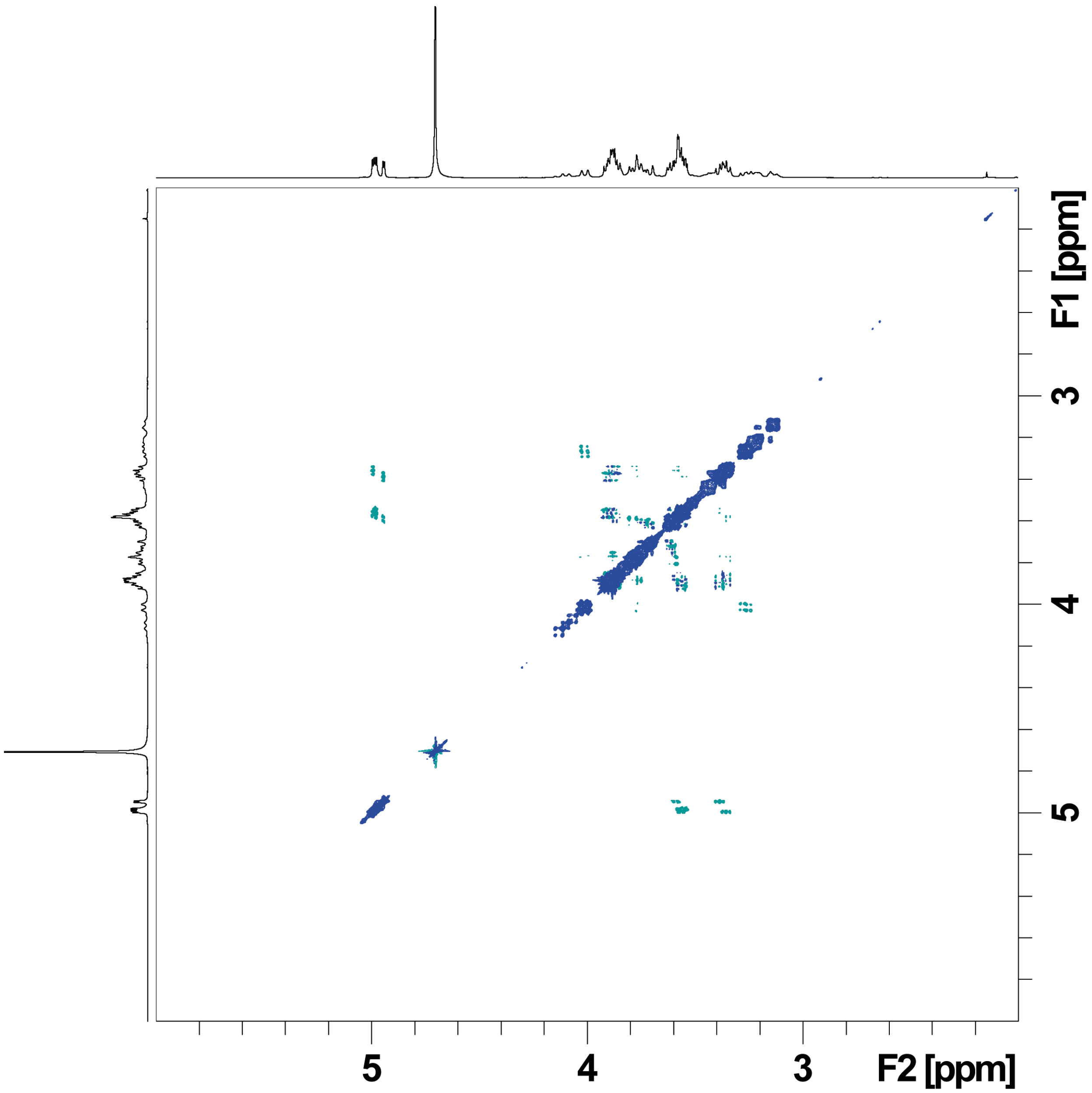

Supplementary Figure 18. ^{1}H-^{1}H **ROESY spectrum (500.08 MHz,** D_2O**) for Cyclodextrin diethylenetriaminepentaacetic acid (CD-DTPA).**

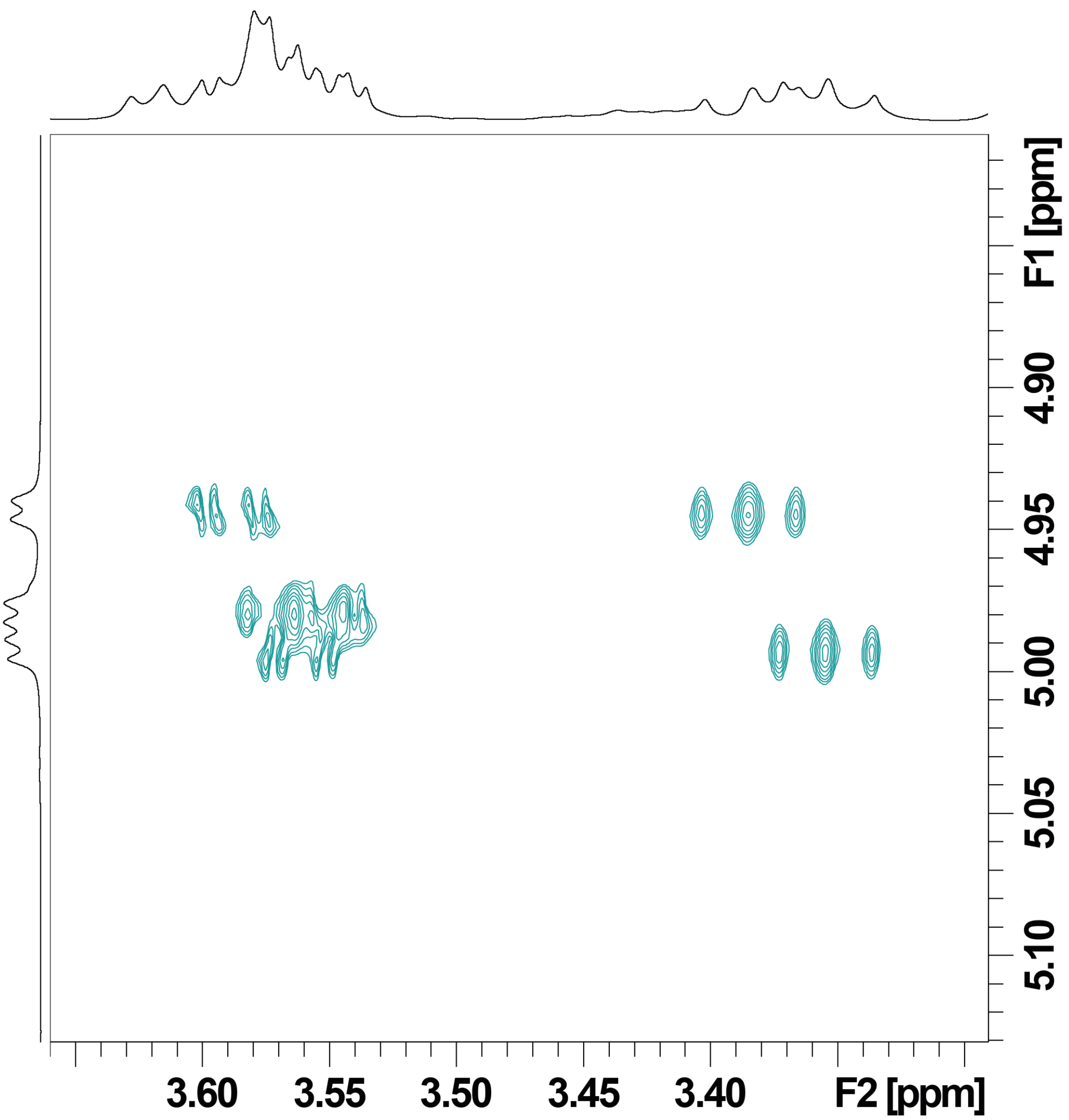

Supplementary Figure 19. Region of ^{1}H-^{1}H ROESY spectrum (Supplementary Figure 18) showing H-1 (F1) and H-4 (F2) correlations.

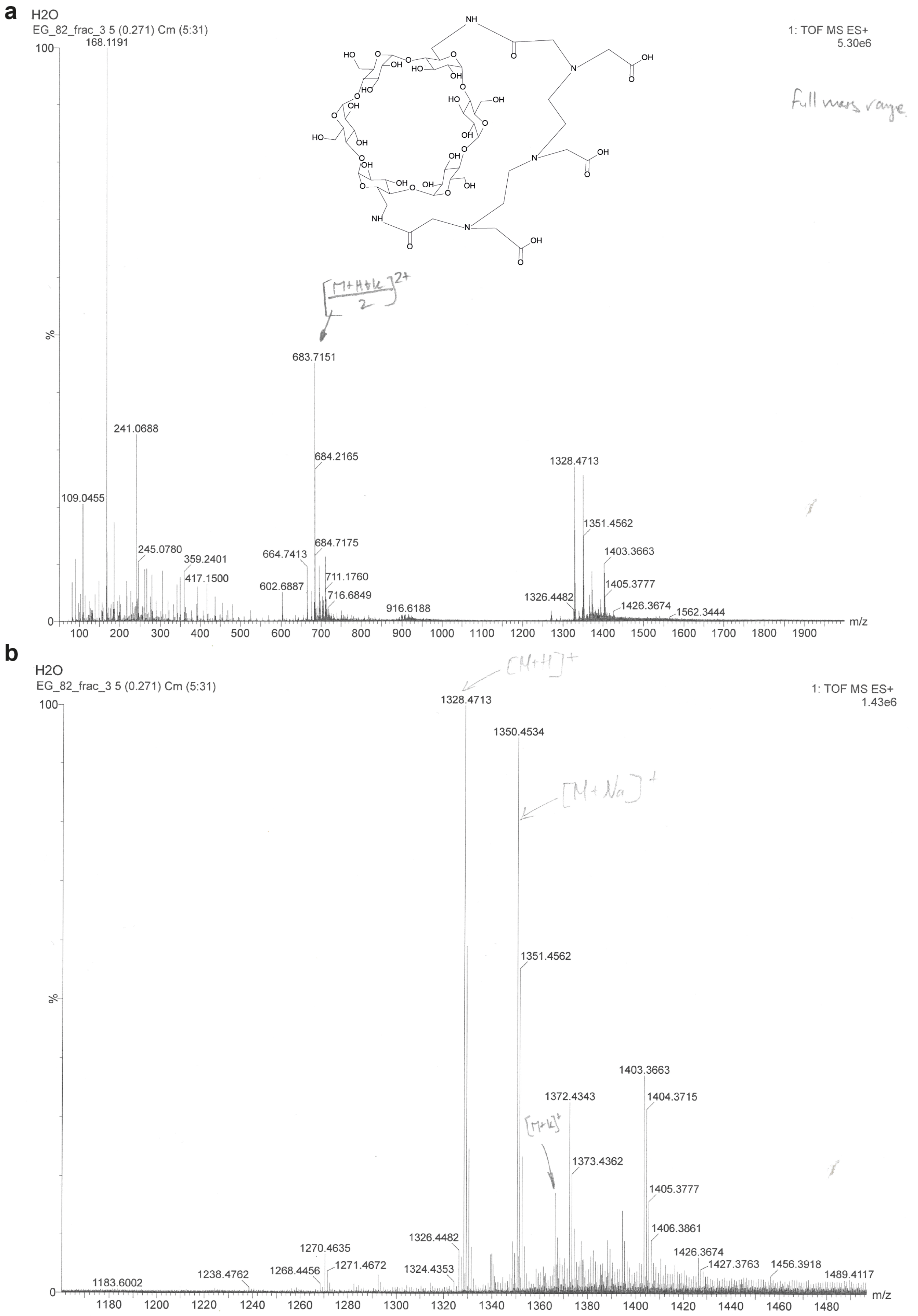

Supplementary Figure 20. MS spectra for Cyclodextrin diethylenetriaminepentaacetic acid (CD-DTPA). (a) Full range spectrum; (b) $[M]^+$ peak range.

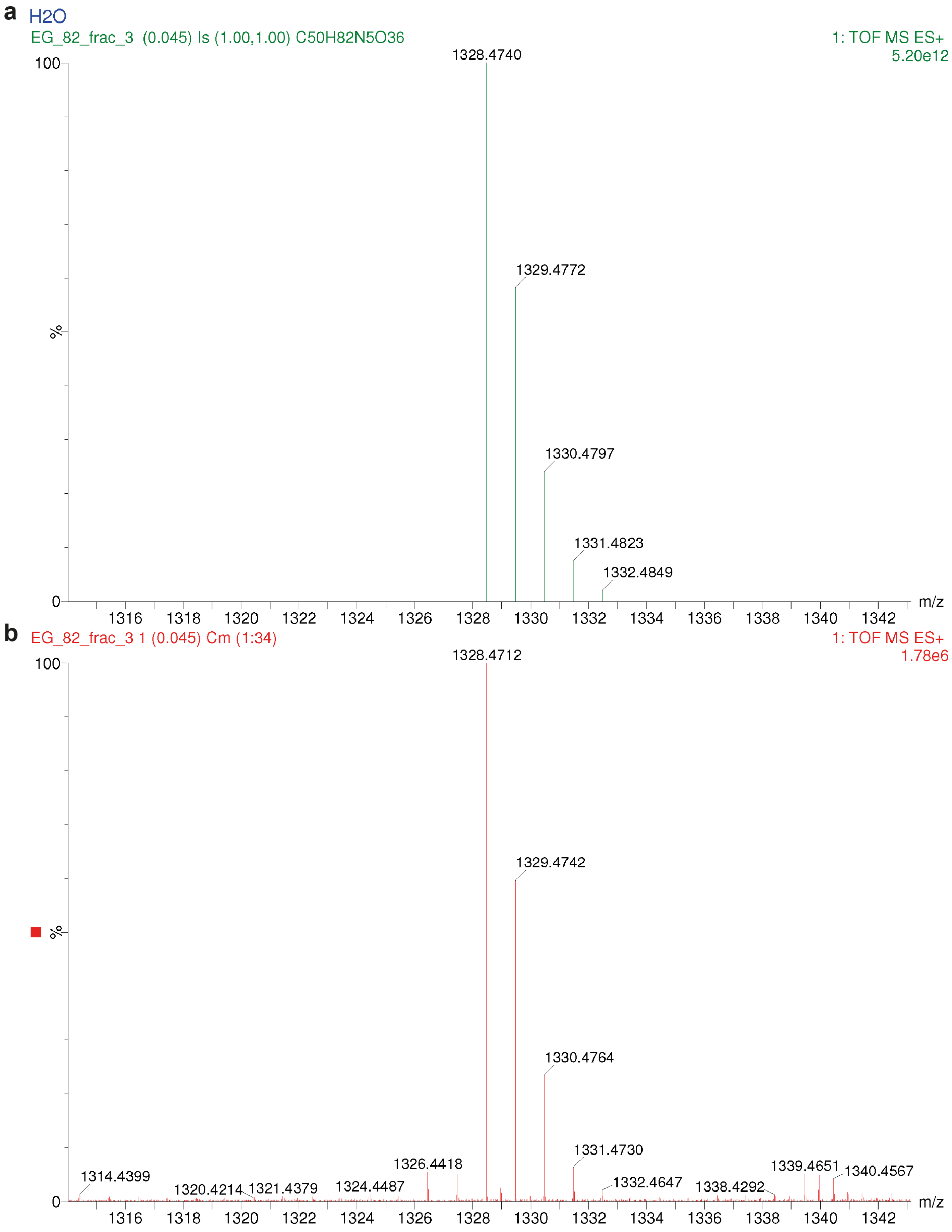

Supplementary Figure 21. Isotopic Patterns for Cyclodextrin diethylenetriaminepentaacetic acid (CD-DTPA). (a) Simulated mass distribution; (b) Experimental mass distribution.

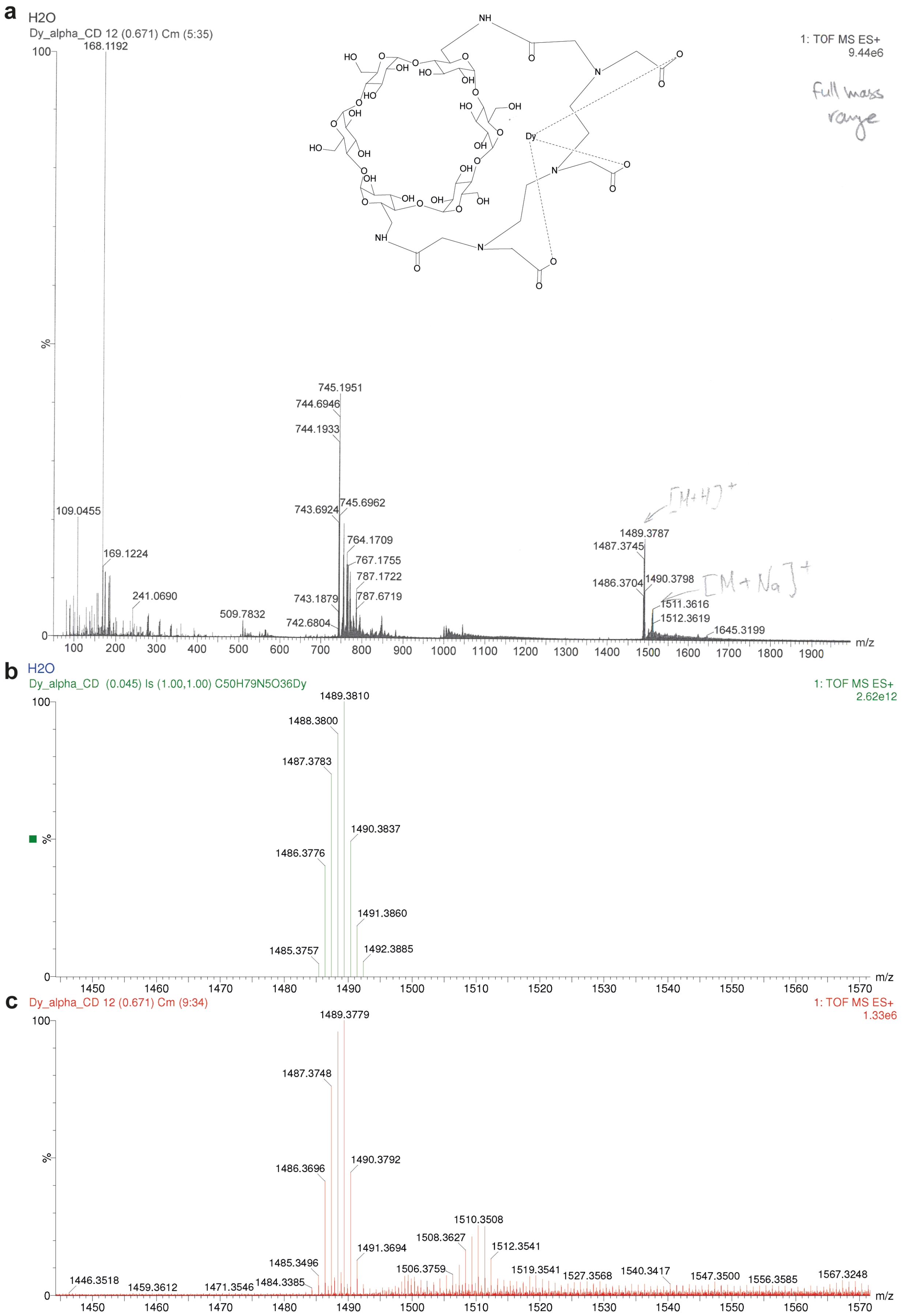

Supplementary Figure 22. MS spectrum and isotopic patterns for Dysprosium-Cyclodextrin (Dy-CD). (a) Full range MS chromatogram; (b) Simulated mass distribution; (c) Experimental mass distribution.

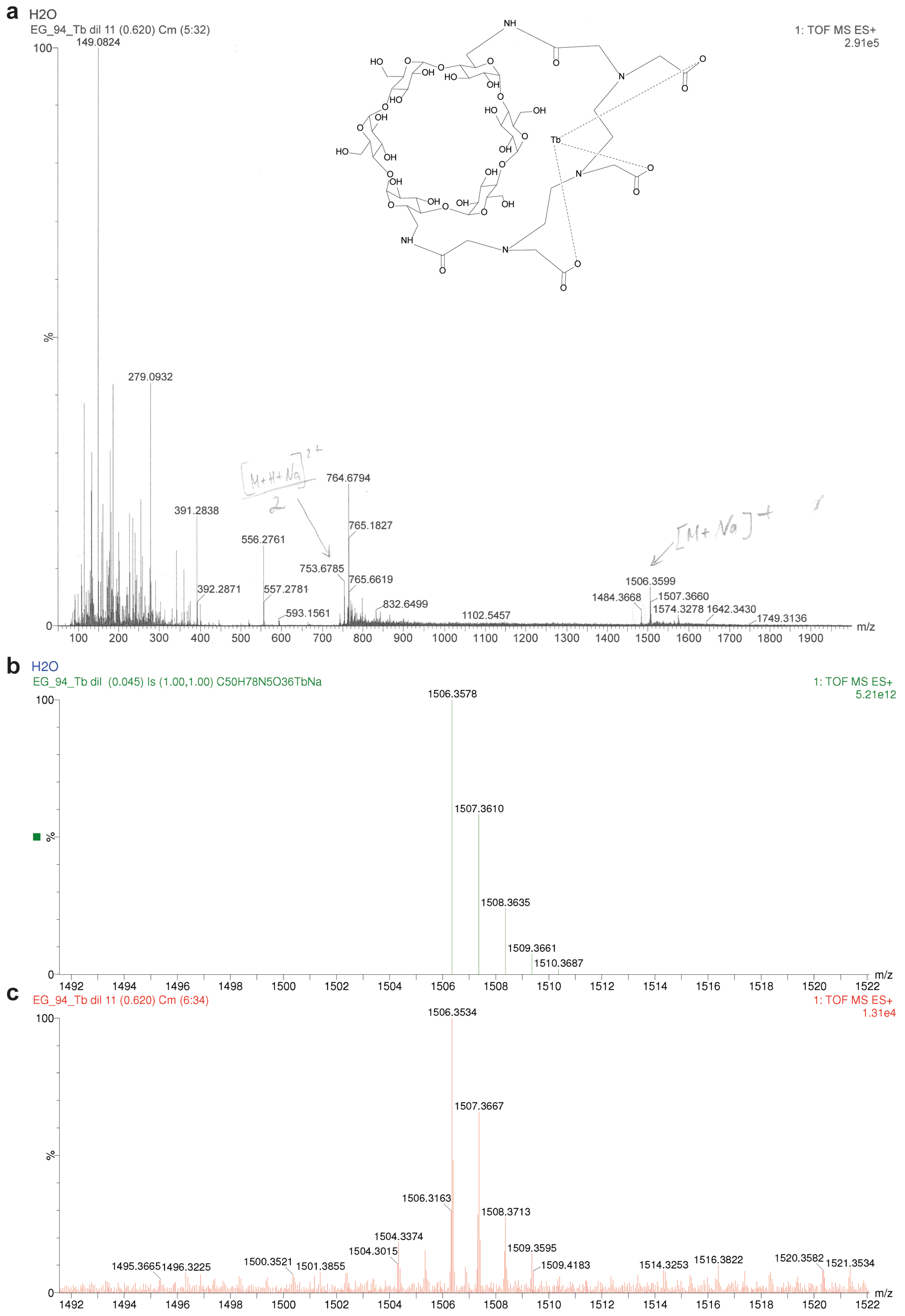

Supplementary Figure 23. MS spectrum and isotopic patterns for Terbium-Cyclodextrin (Tb-CD). (a) Full range MS chromatogram; (b) Simulated mass distribution; (c) Experimental mass distribution.

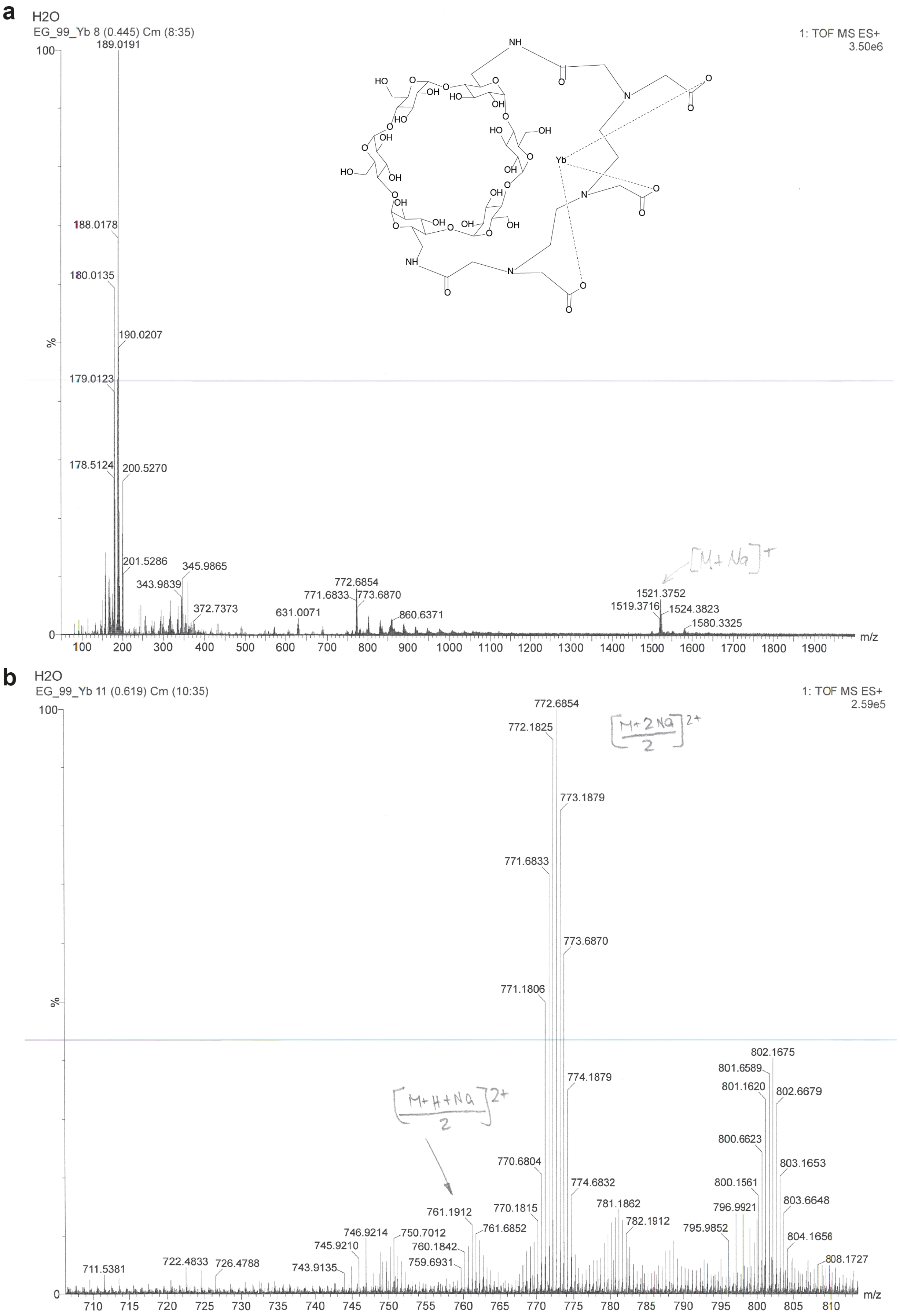

Supplementary Figure 24. MS spectrum for Ytterbium-Cyclodextrin (Yb-CD). (a) Full range spectrum; (b) zoom for $[M]^{+2}$ peak range.

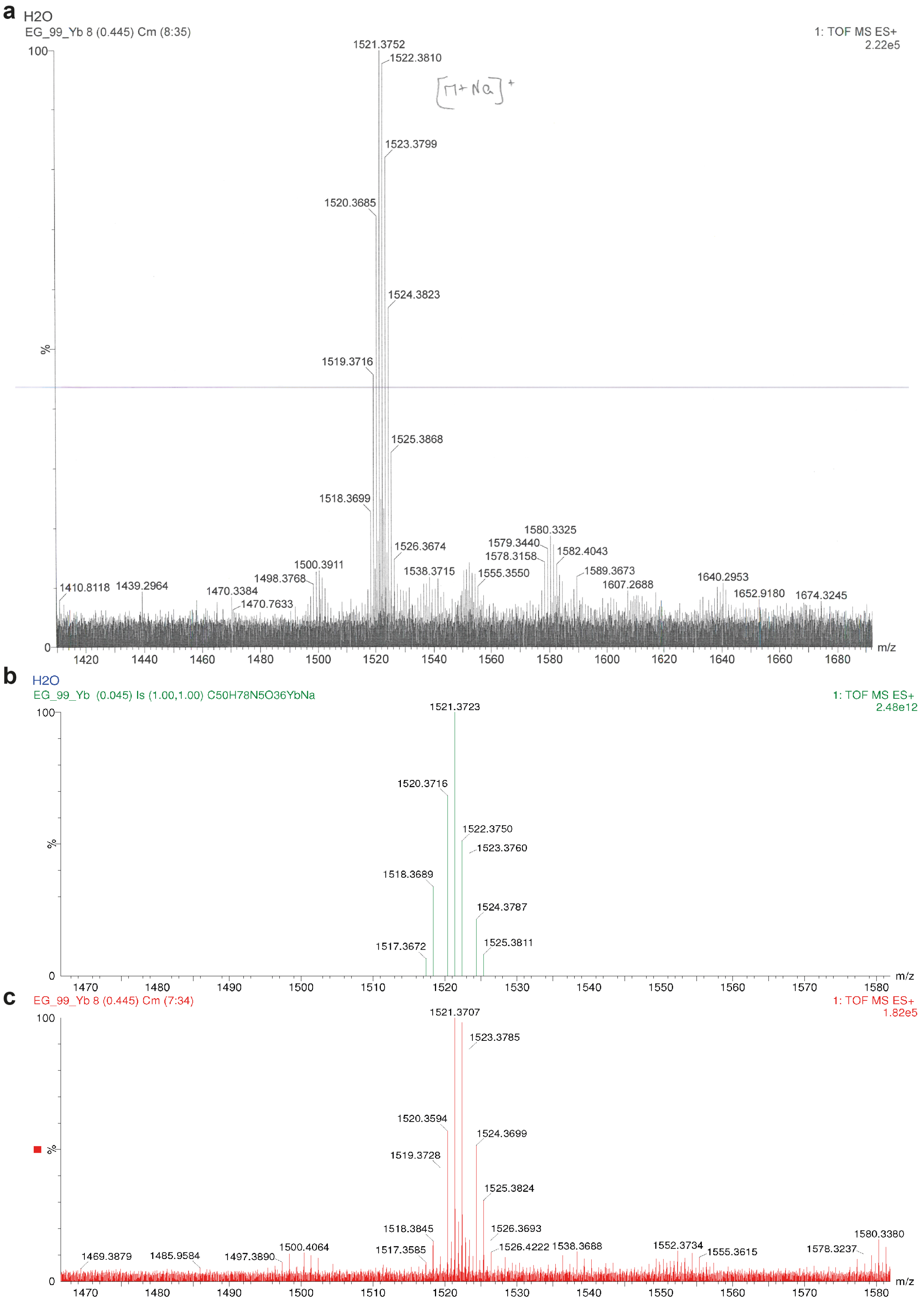

Supplementary Figure 25. MS spectrum and isotopic patterns for Ytterbium-Cyclodextrin (Yb-CD). (a) Zoom for $[M]^+$ peak range.; (b) Simulated mass distribution; (c) Experimental mass distribution.

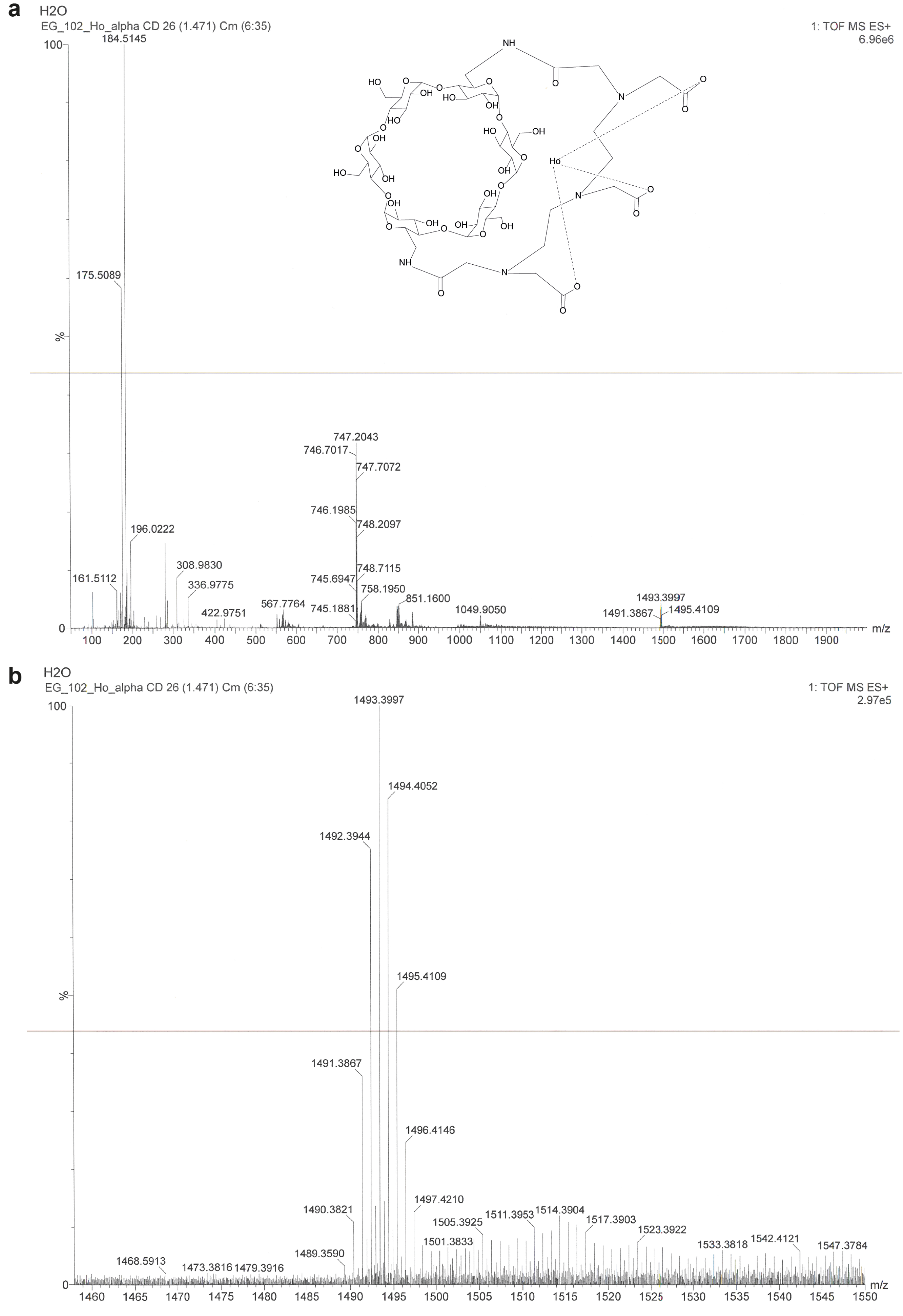

Supplementary Figure 26. MS spectrum for Holmium-Cyclodextrin (Ho-CD). (a) Full range spectrum; (b) Zoom for $[M]^+$ peak range.

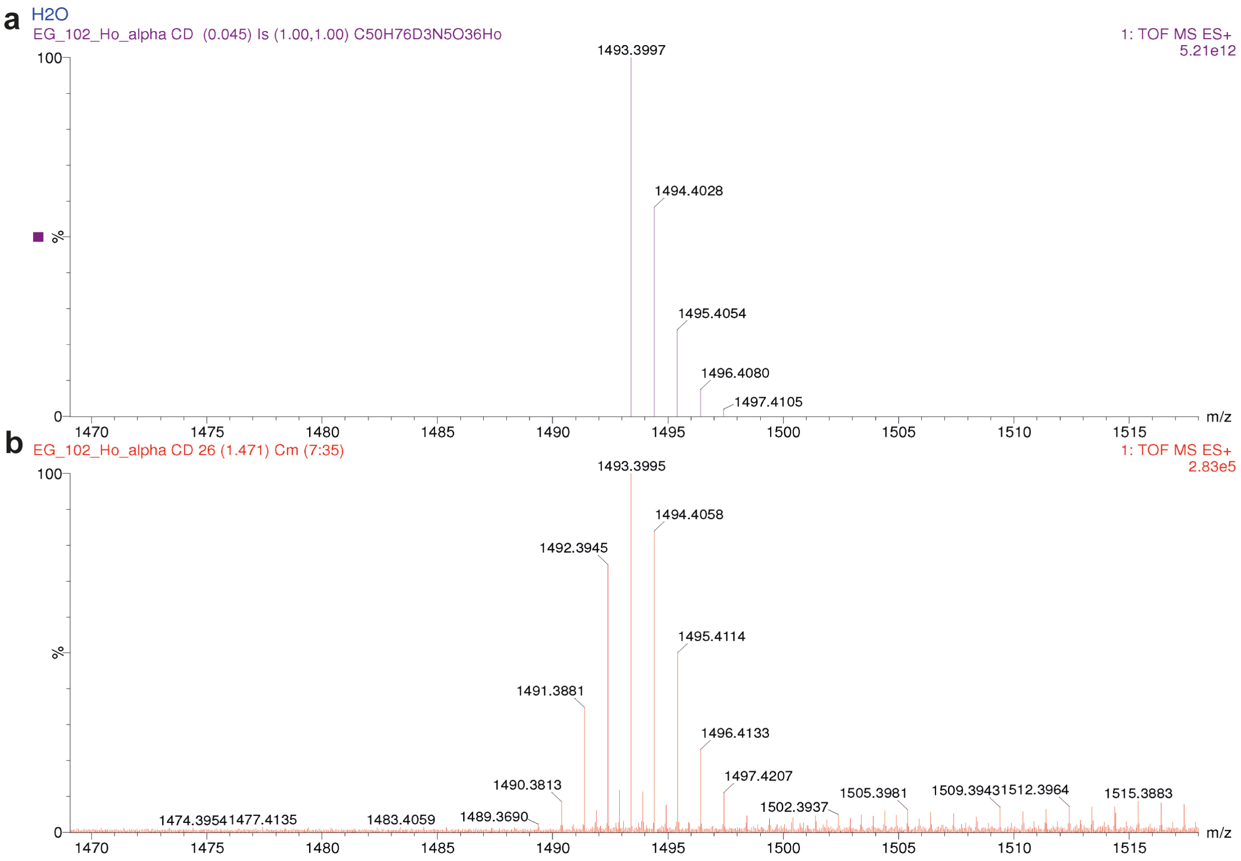

Supplementary Figure 27. Isotopic Patterns for Holmium-Cyclodextrin (Ho-CD). (a) Simulated mass distribution; (b) Experimental mass distribution.

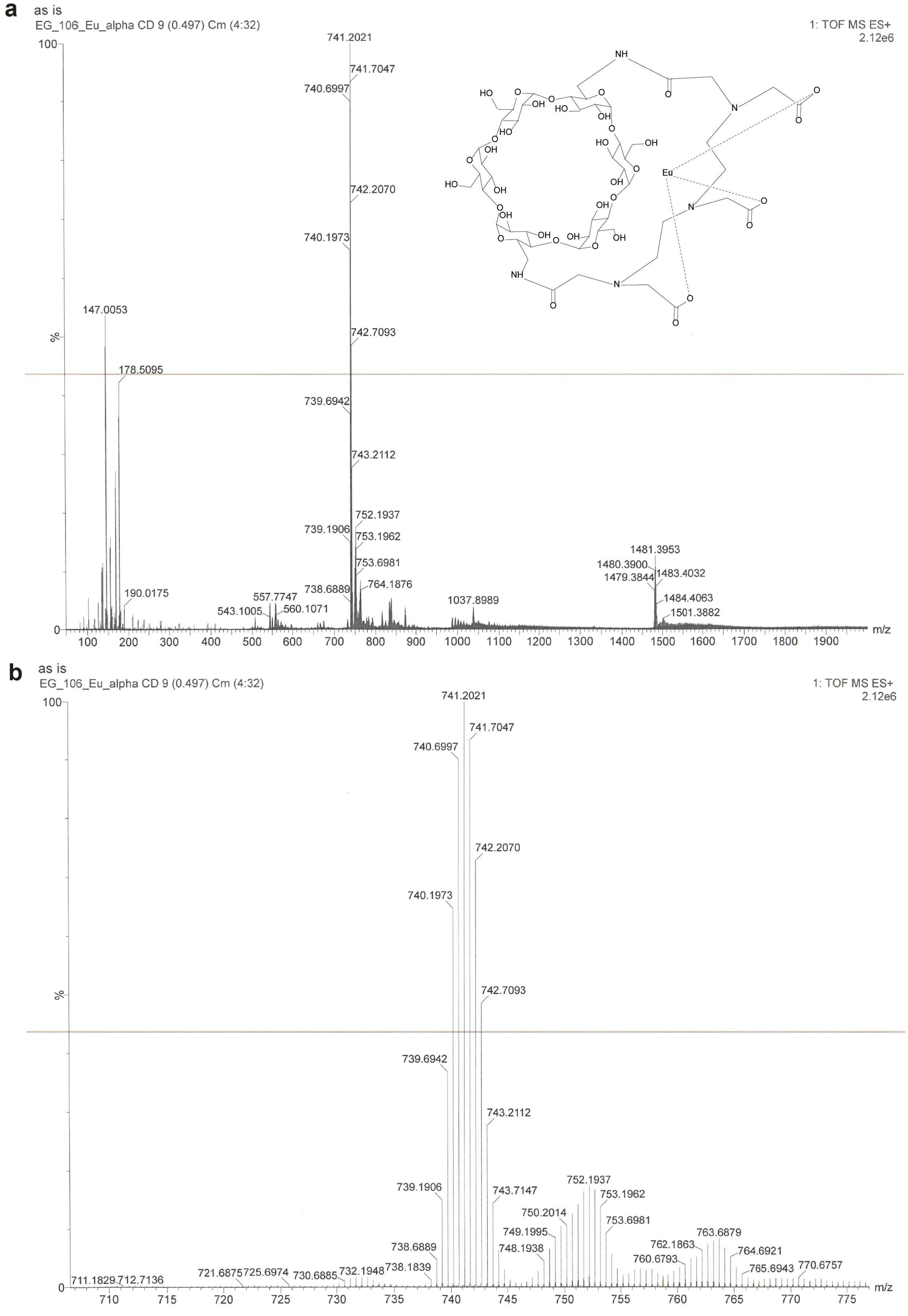

Supplementary Figure 28. MS spectra for Europium-Cyclodextrin (Eu-CD). (a) Full range spectrum; (b) Zoom for $[M]^{+2}$ peak range.

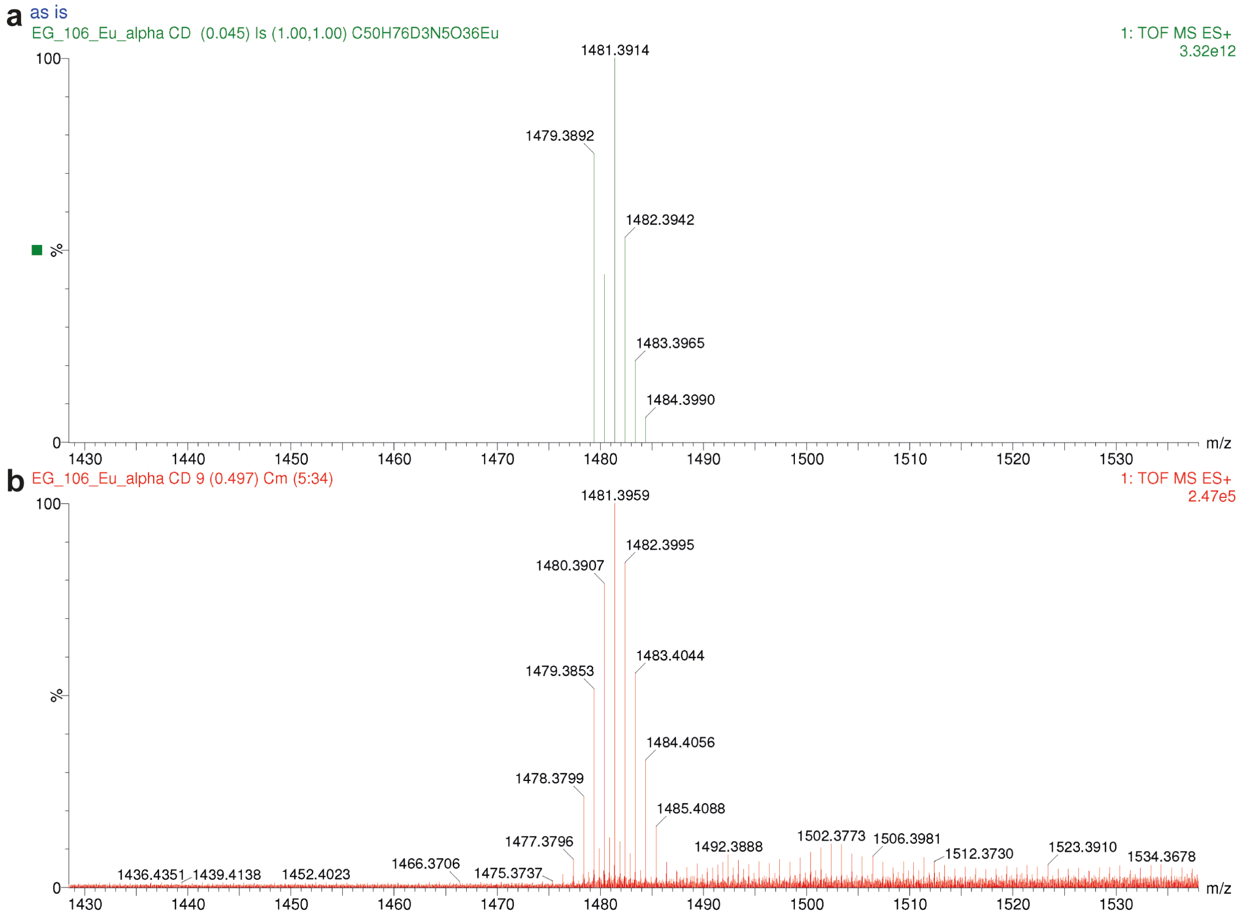

Supplementary Figure 29. Isotopic Patterns for Europium-Cyclodextrin (Eu-CD). (a) Simulated mass distribution; (b) Experimental mass distribution.

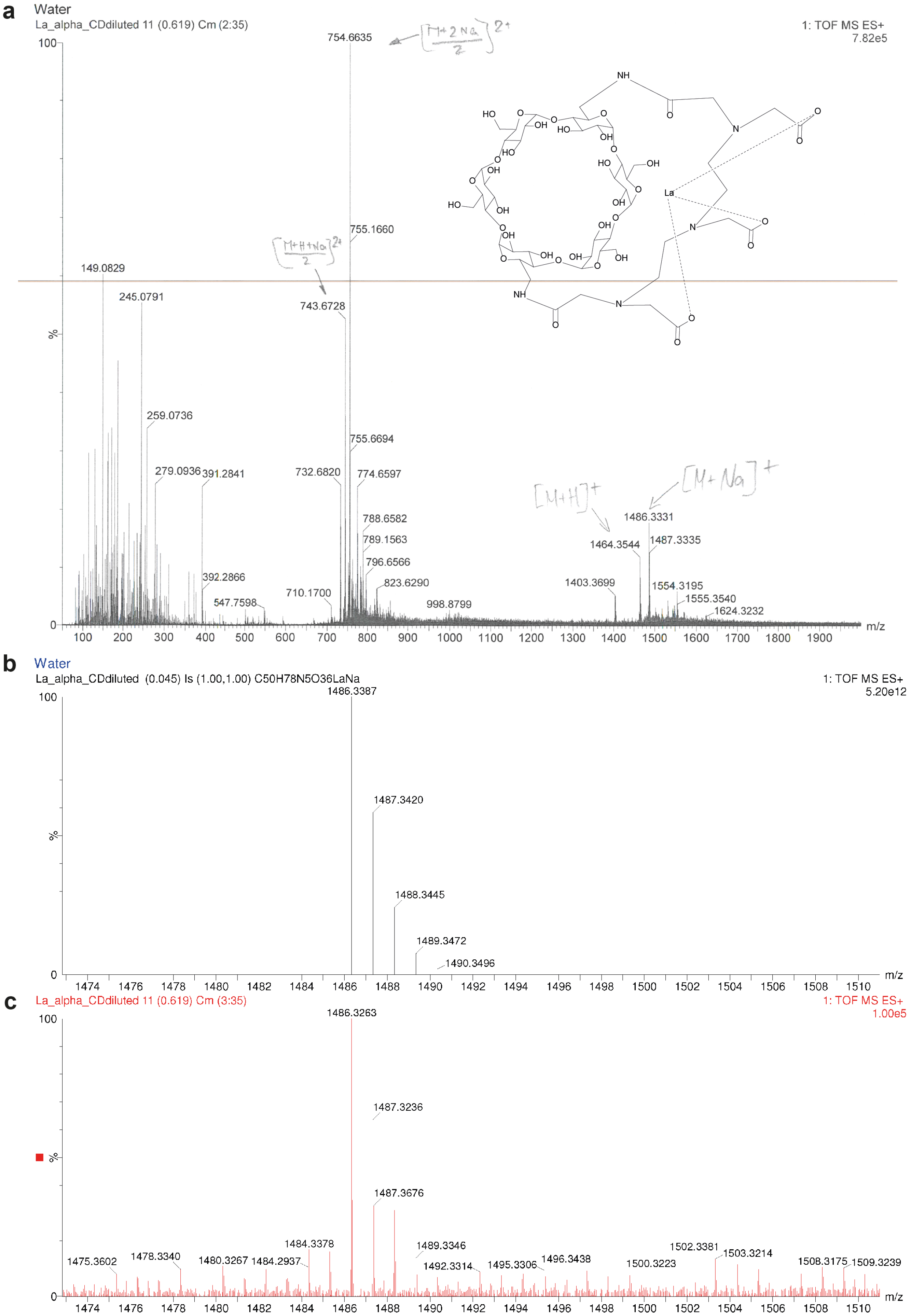

Supplementary Figure 30. MS spectrum and isotopic patterns for Lanthanum-Cyclodextrin (La-CD). (a) Full range MS chromatogram; (b) Simulated mass distribution; (c) Experimental mass distribution.

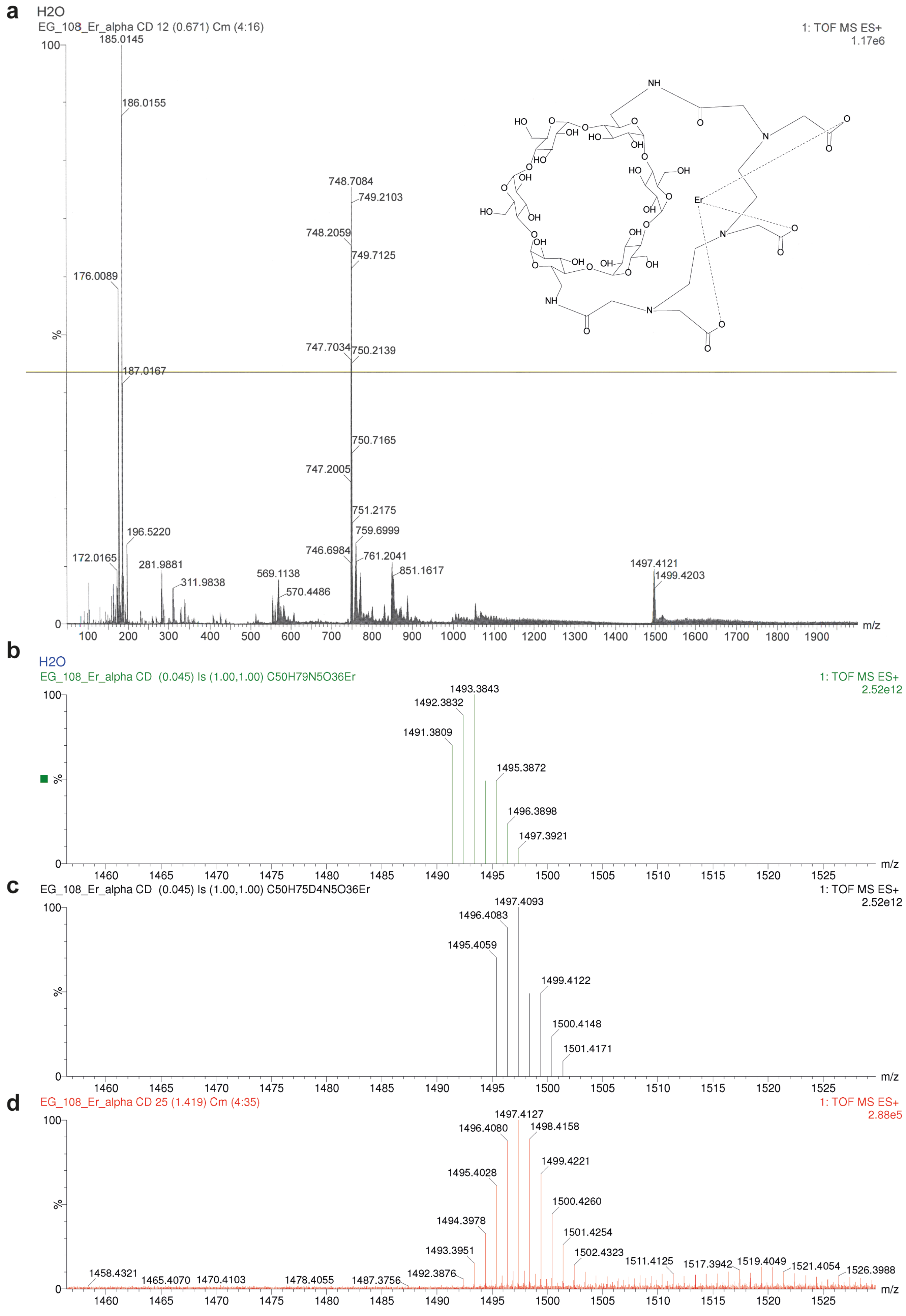

Supplementary Figure 31. MS spectrum and isotopic patterns for Erbium-Cyclodextrin (Er-CD). (a) Full range MS chromatogram (D_2O); (b) Simulated mass distribution (H_2O); (c) Simulated mass distribution (D_2O); (d) Experimental mass distribution.

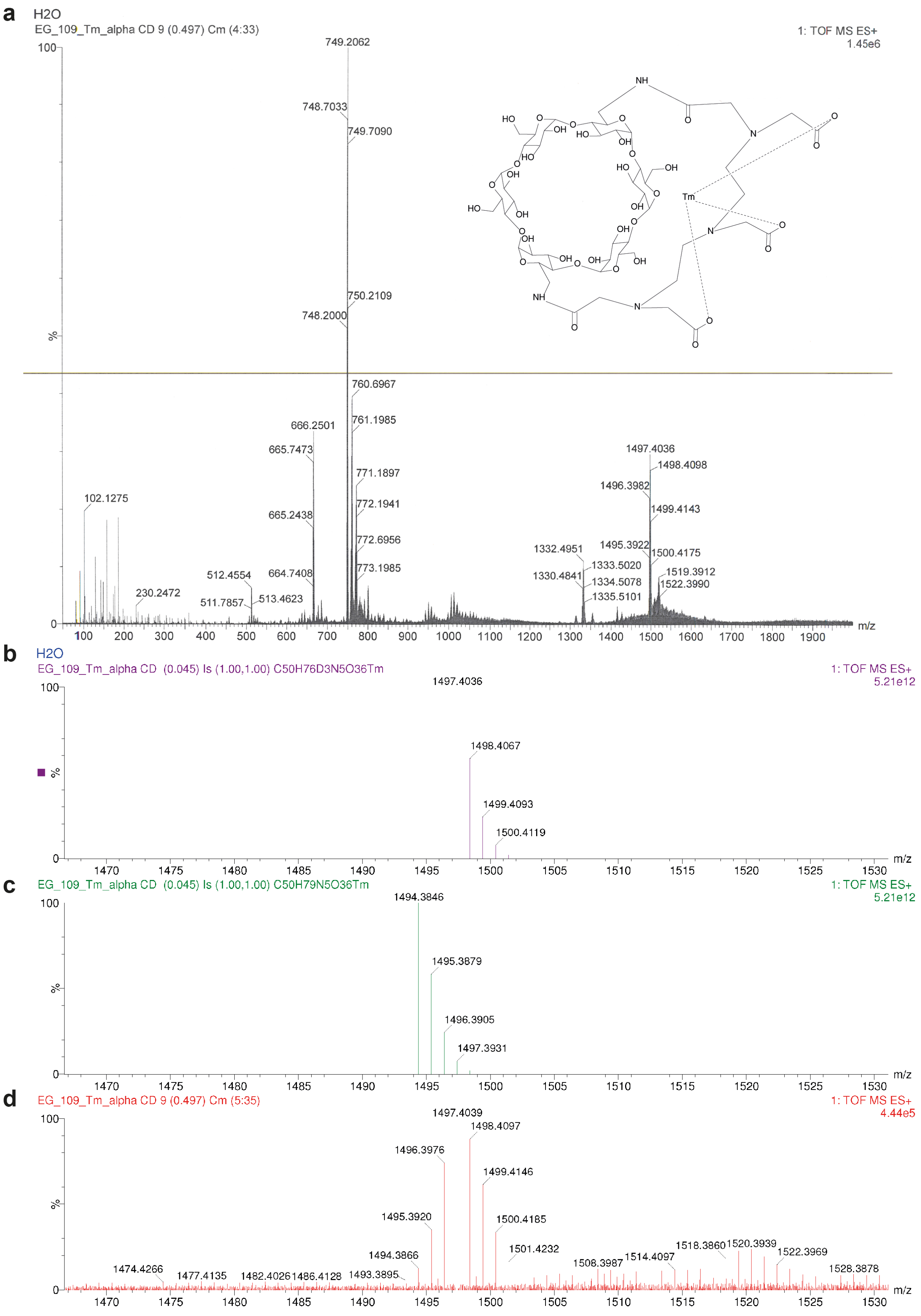

Supplementary Figure 32. MS spectrum and isotopic patterns for Thulium-Cyclodextrin (Tm-CD). (a) Full range MS chromatogram (D_2O); (b) Simulated mass distribution (D_2O); (c) Simulated mass distribution (H_2O); (d) Experimental mass distribution.

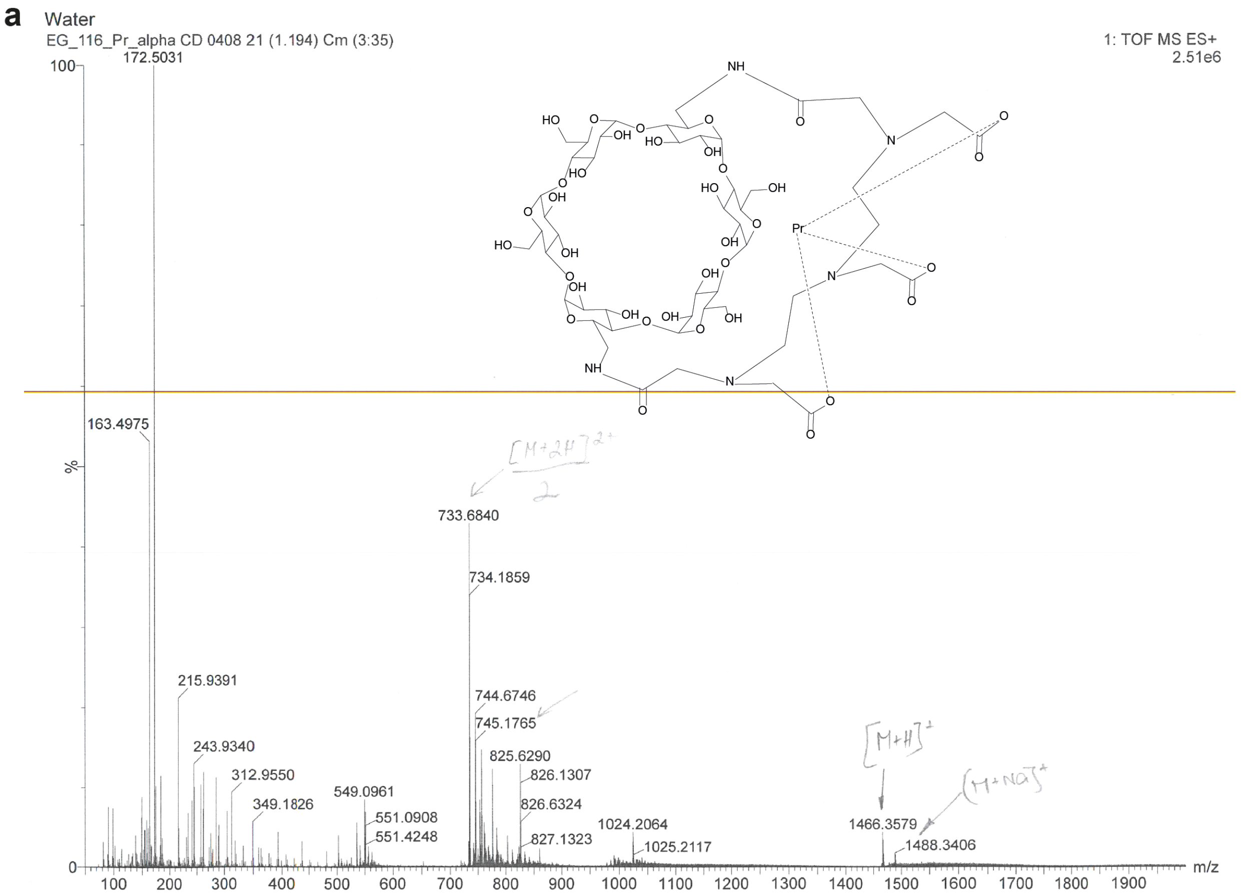

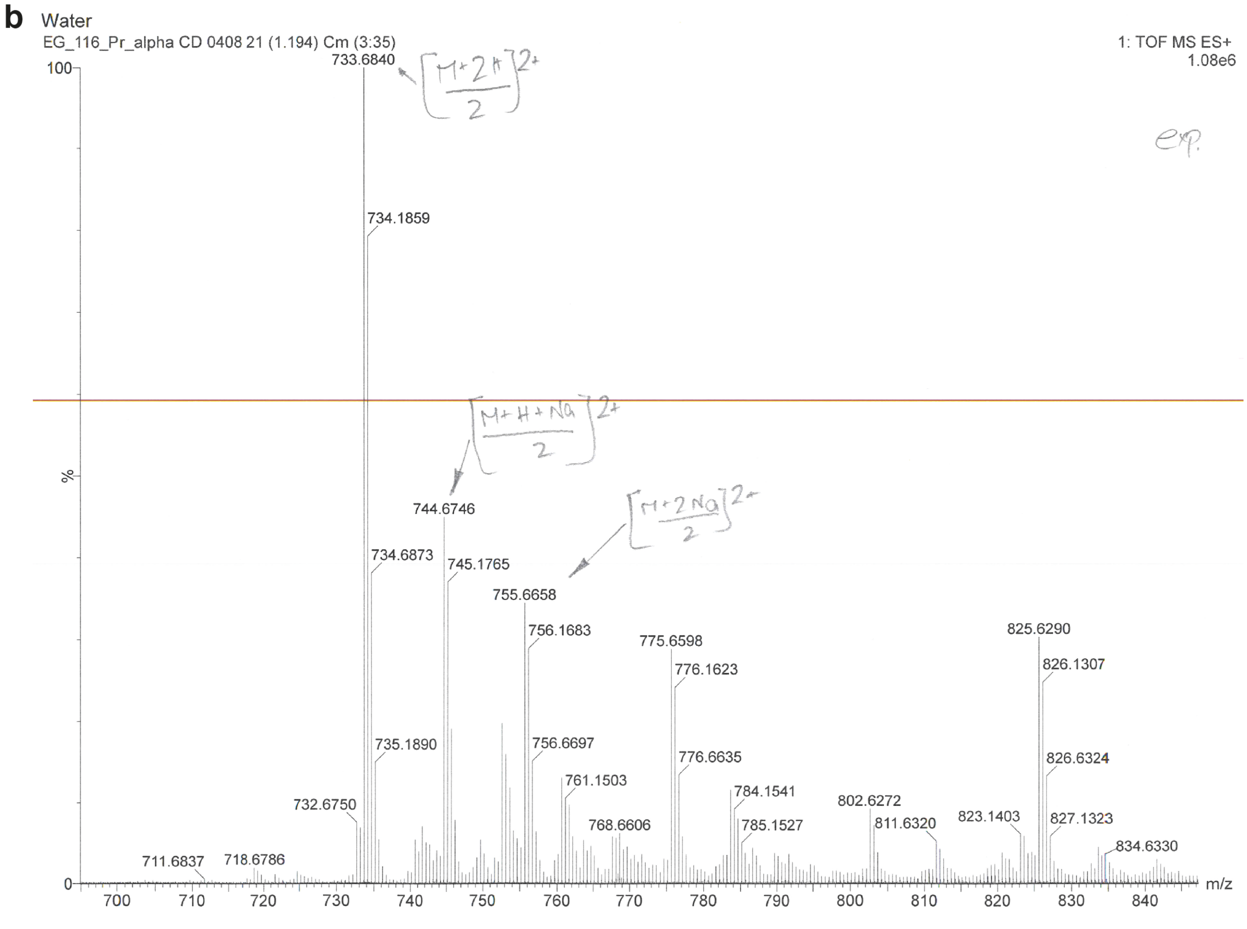

Supplementary Figure 33. MS spectra for Praseodymium-Cyclodextrin (Pr-CD). (a) Full range spectrum; (b) Zoom for $[M]^{+2}$ peak range.

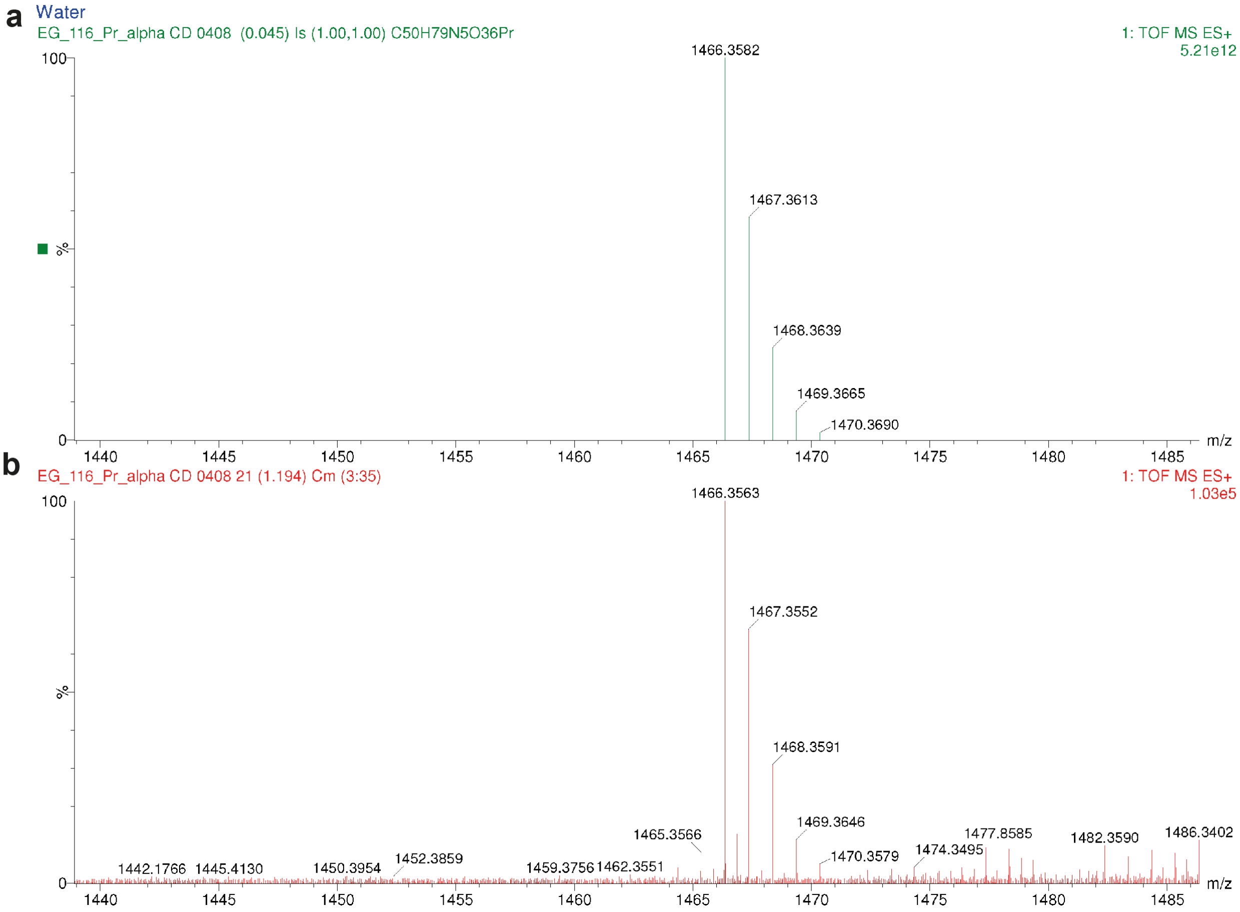

Supplementary Figure 34. Isotopic Patterns for Praseodymium-Cyclodextrin (Pr-CD). (a) Simulated mass distribution; (b) Experimental mass distribution.

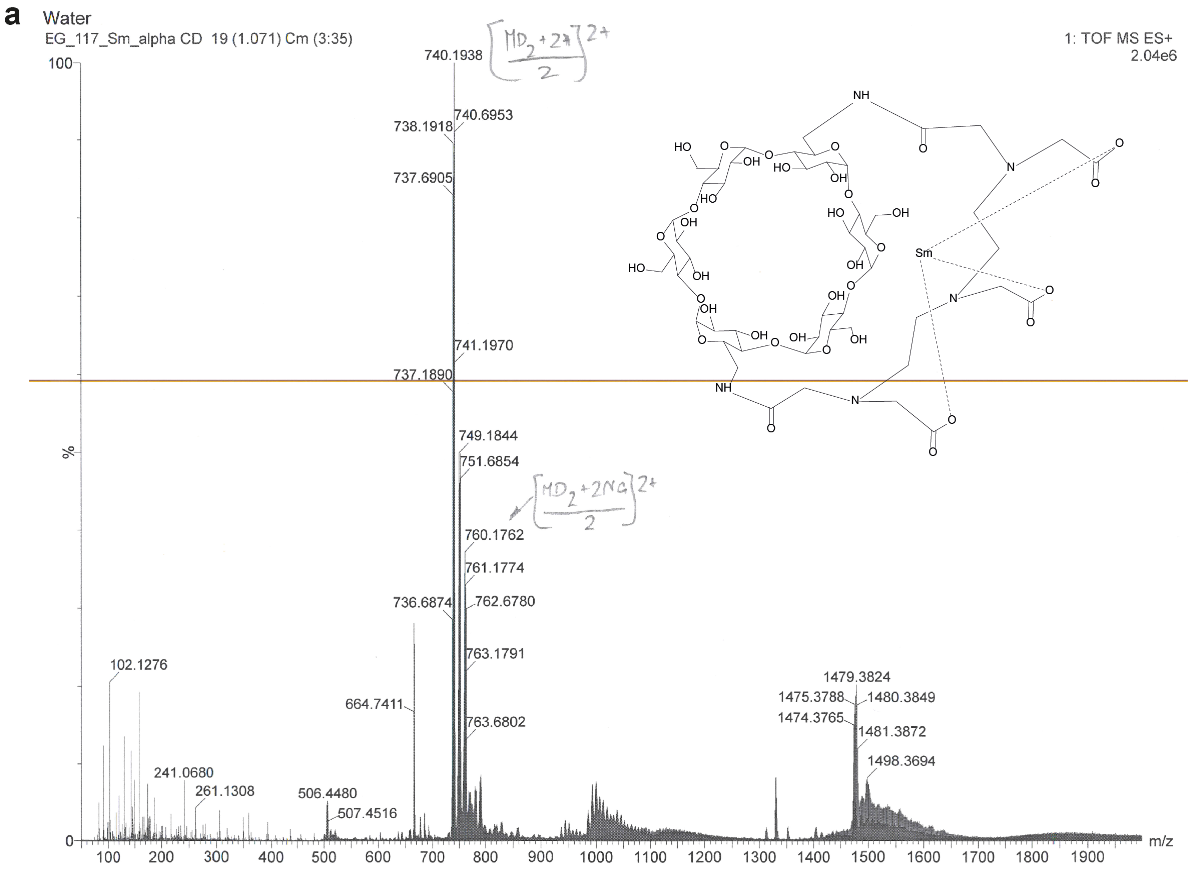

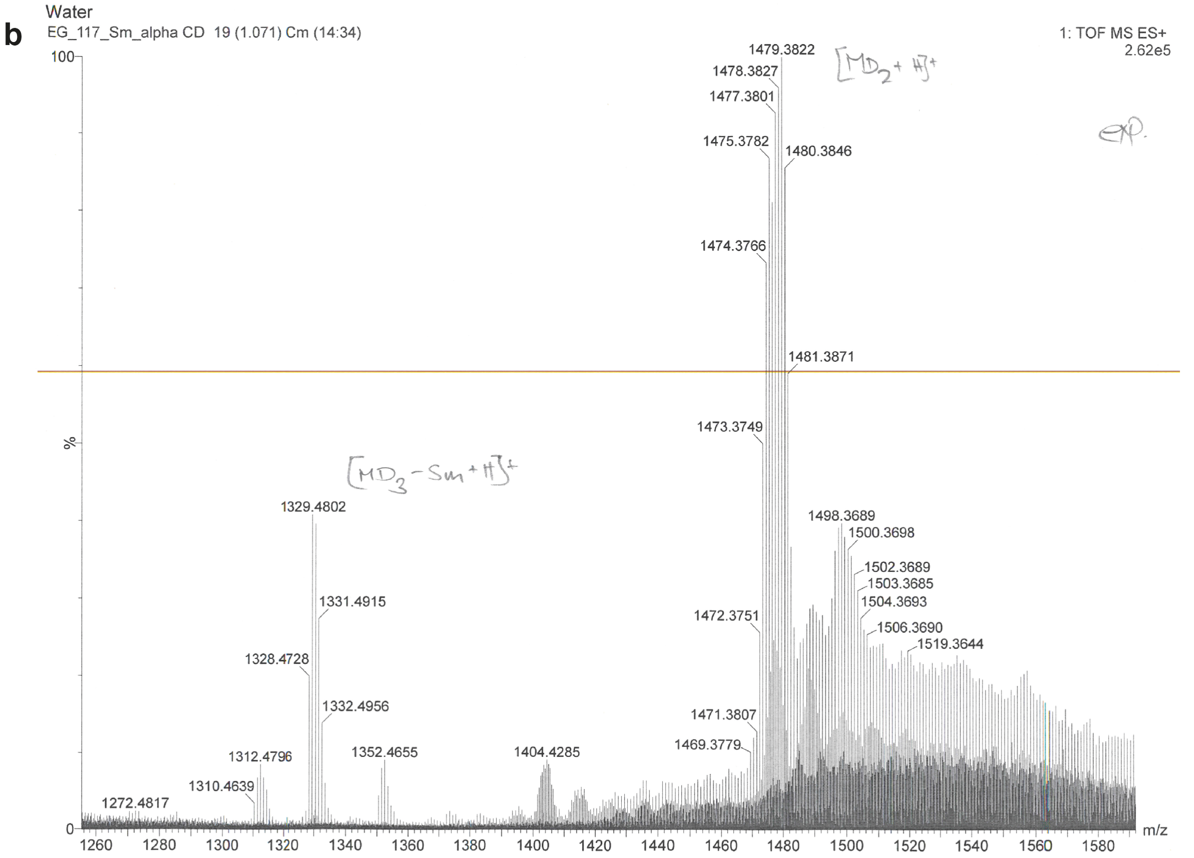

Supplementary Figure 35. MS spectra for Samarium-Cyclodextrin (Sm-CD). (a) Full range spectrum (D_2O); (b) Zoom for $[M]^+$ peak range.

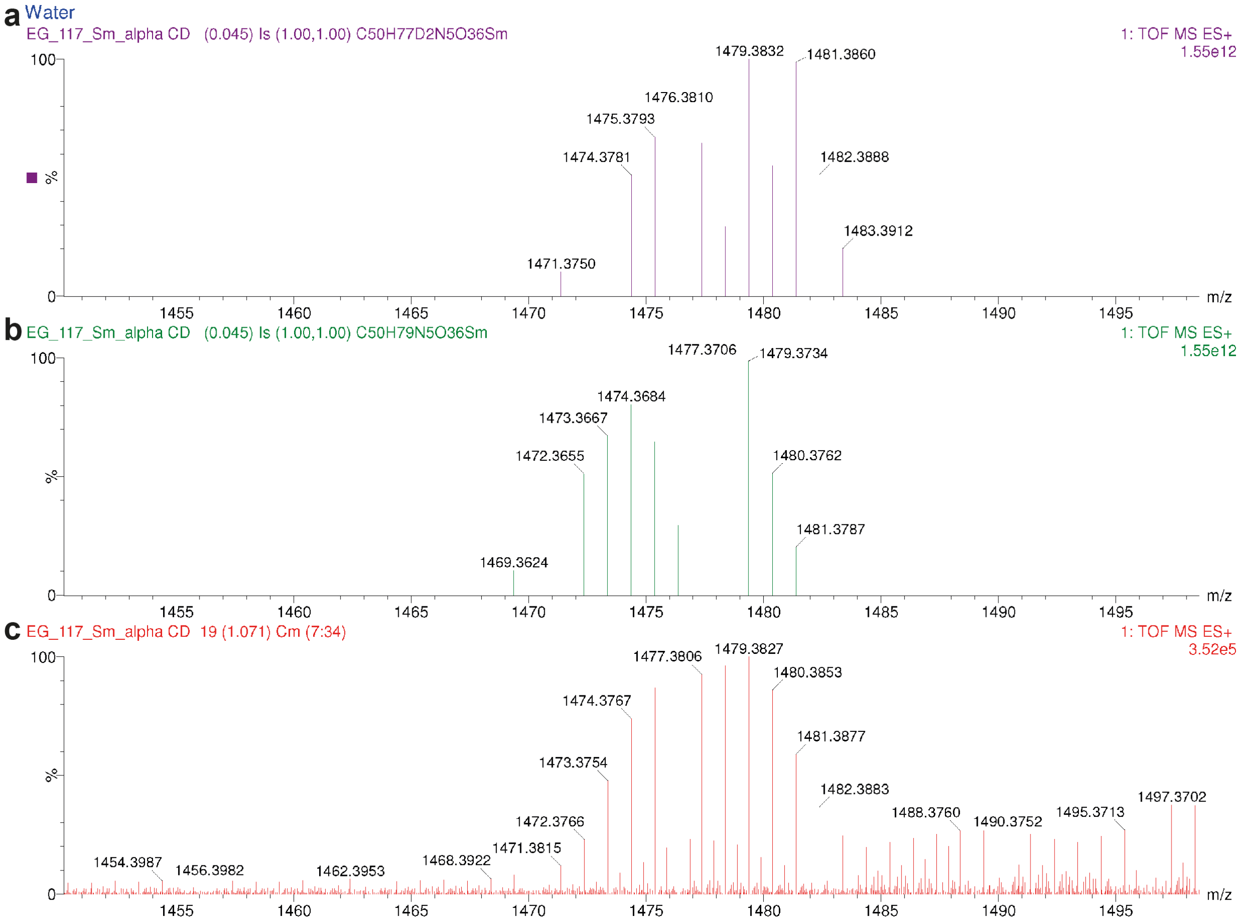

Supplementary Figure 36. Isotopic patterns for Samarium-Cyclodextrin (Sm-CD). (a) Simulated mass distribution (D_2O); (b) Simulated mass distribution (H_2O); (c) Experimental mass distribution.

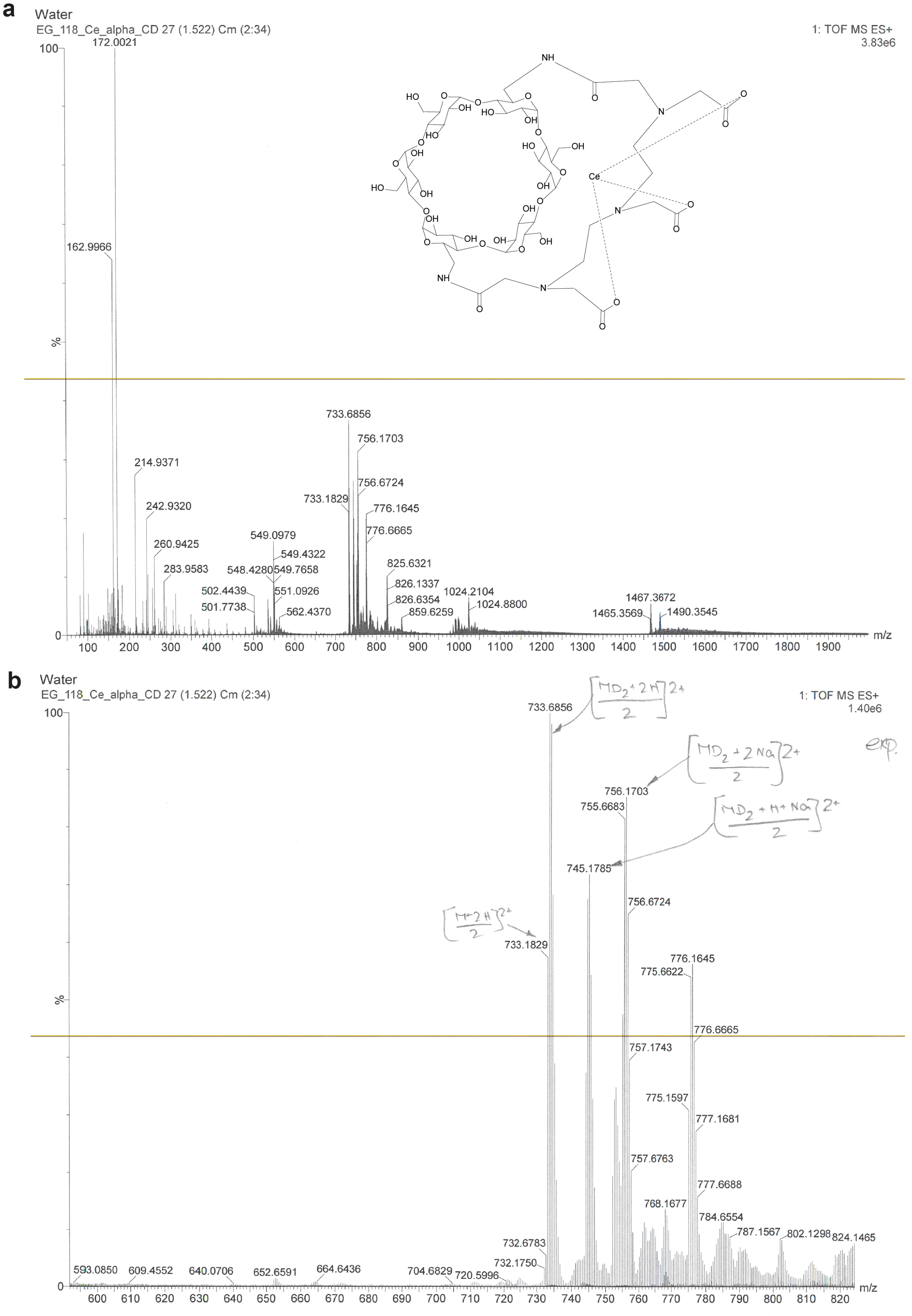

Supplementary Figure 37. MS spectra for Cerium-Cyclodextrin (Ce-CD). (a) Full range spectrum (D_2O); (b) Zoom for $[M]^{+2}$ peak range.

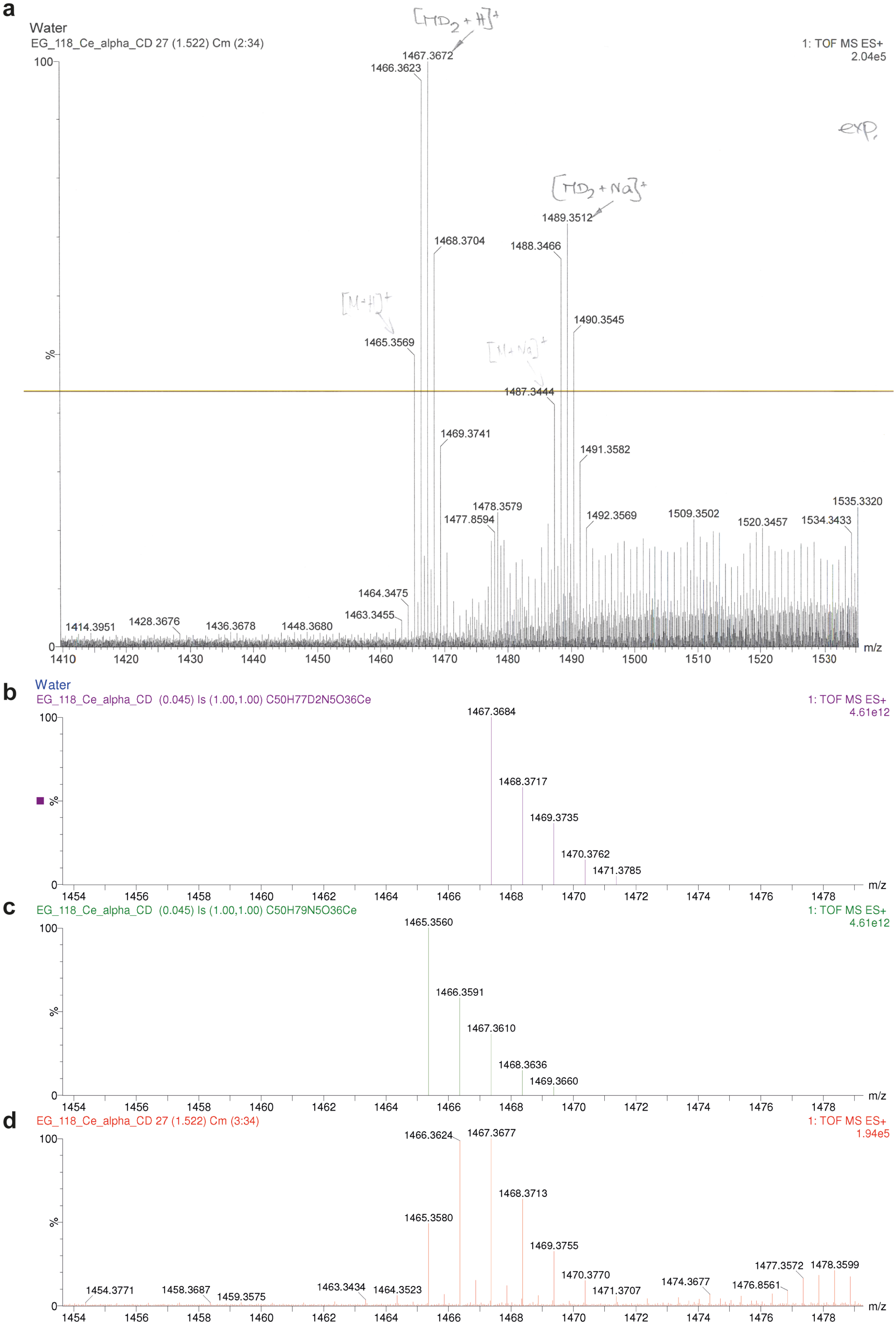

Supplementary Figure 38. MS spectrum and isotopic patterns for Cerium-Cyclodextrin (Ce-CD). (a) Zoom for $[M]^+$ peak range (D_2O); (b) Simulated mass distribution (D_2O); (c) Simulated mass distribution (H_2O); (d) Experimental mass distribution.

a

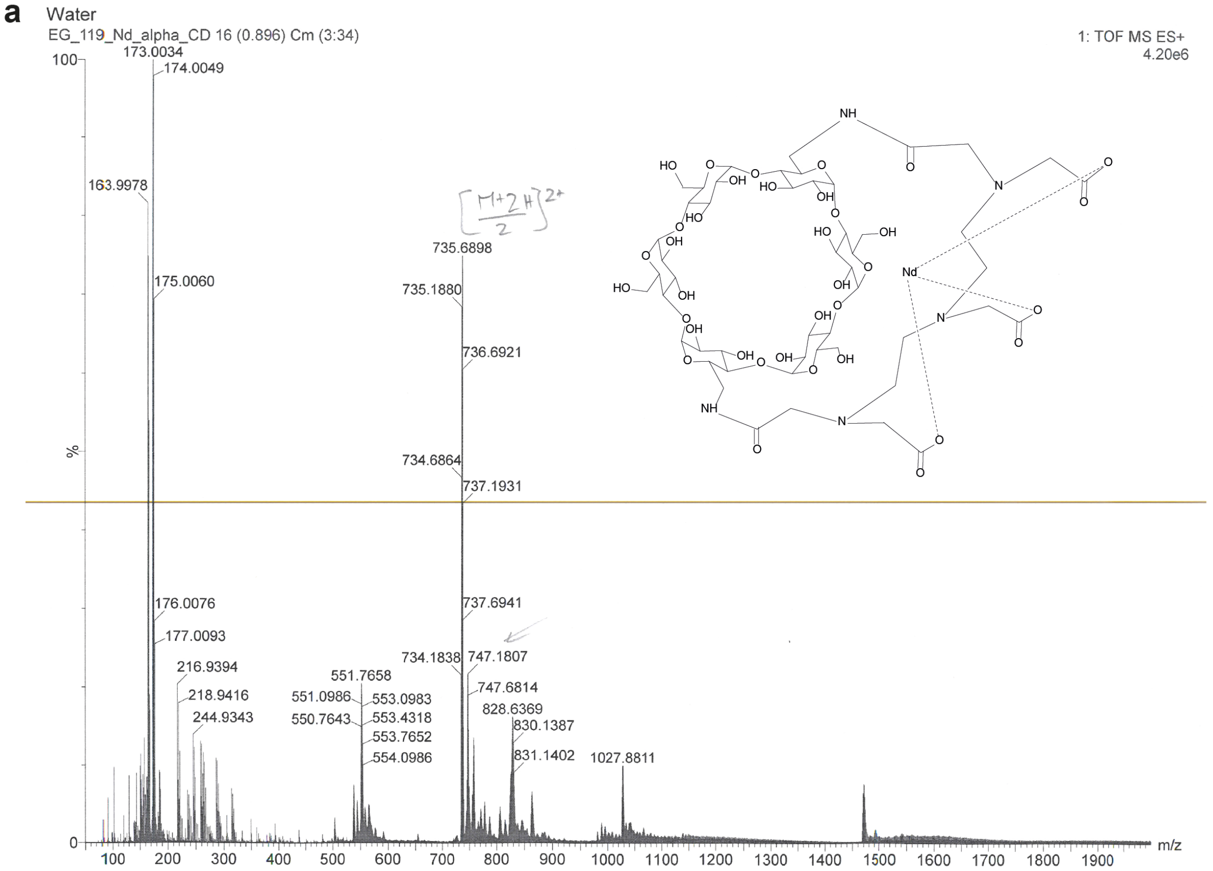

b

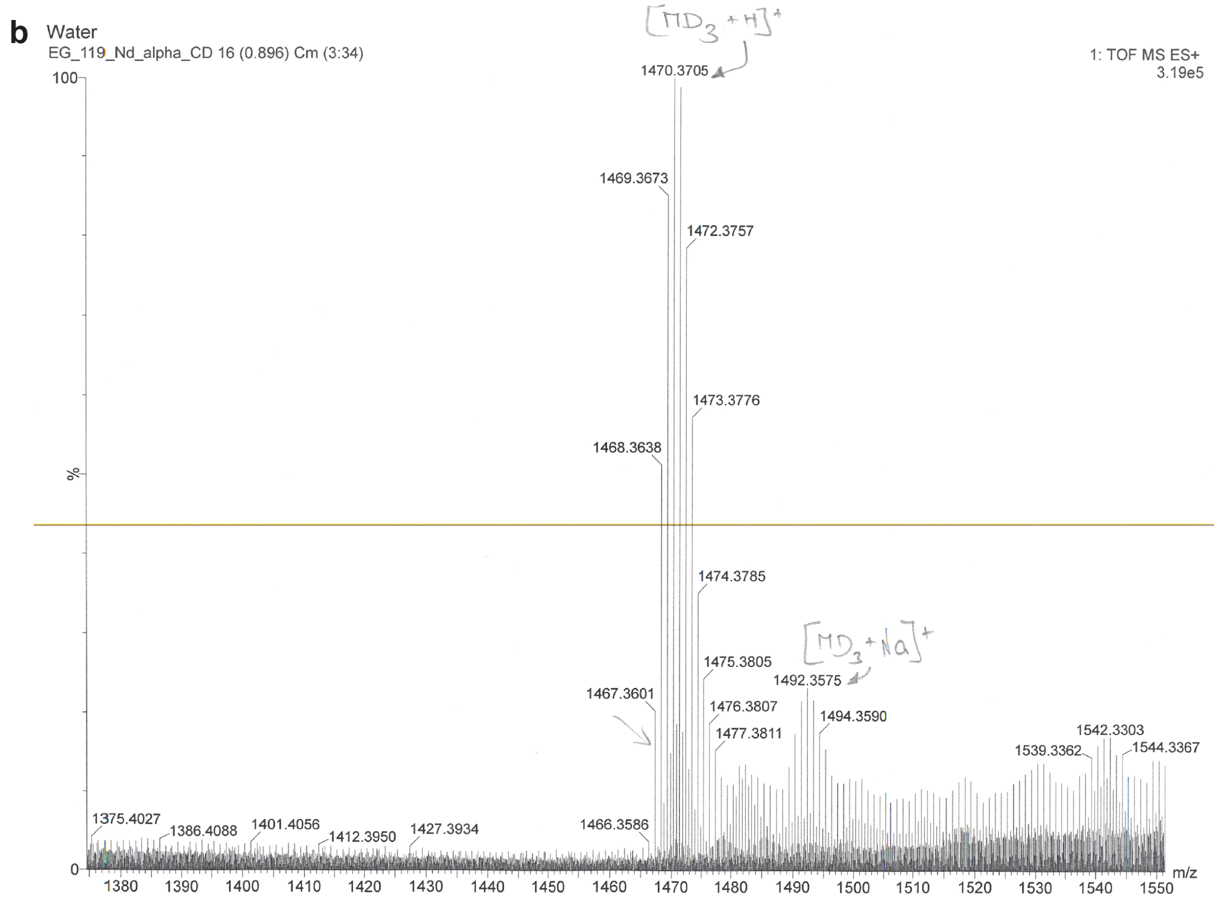

Supplementary Figure 39. MS spectra for Neodymium-Cyclodextrin (Nd-CD). (a) Full range spectrum (D_2O); (b) Zoom for $[M]^+$ peak range.

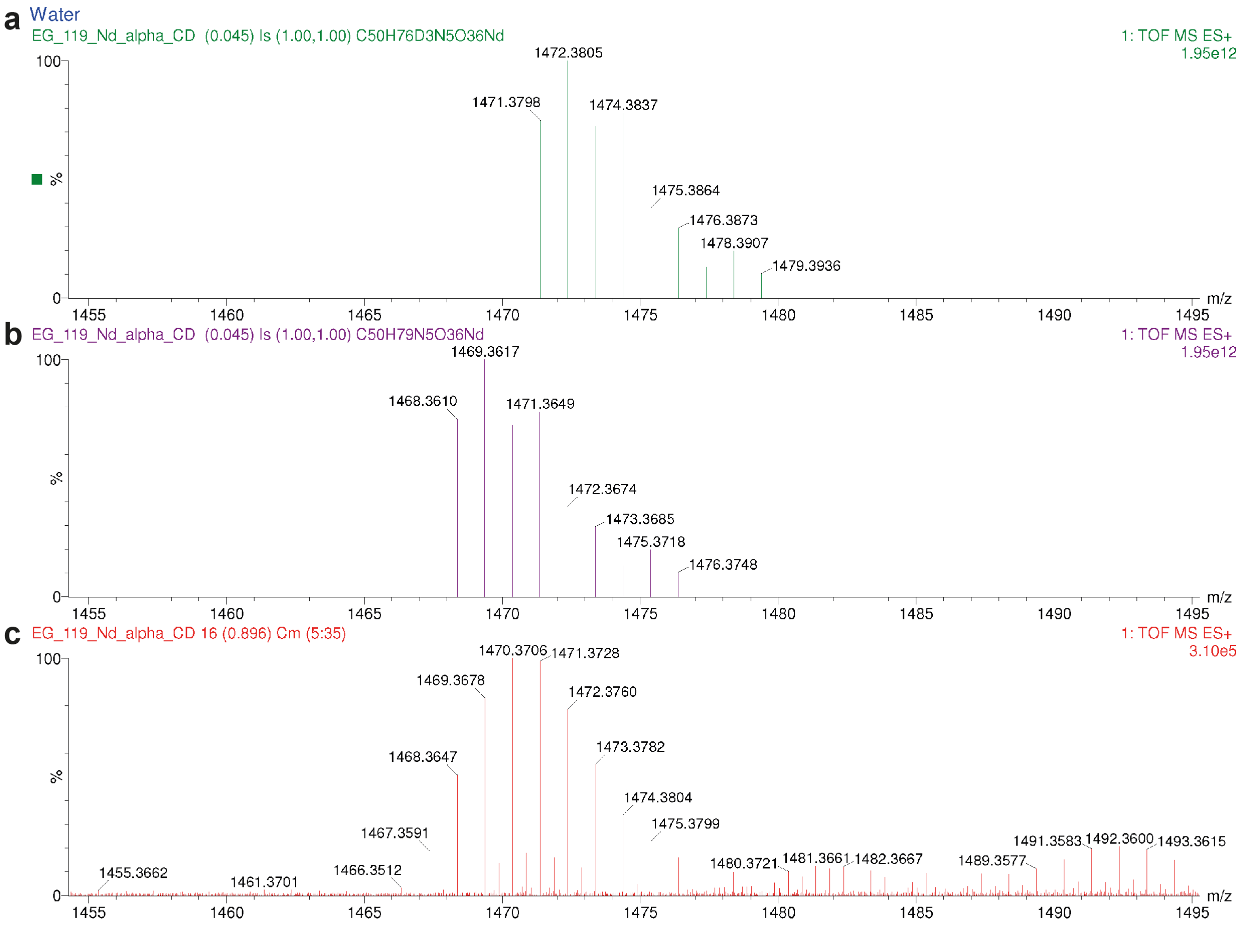

Supplementary Figure 40. Isotopic patterns for Neodymium-Cyclodextrin (Nd-CD). (a) Simulated mass distribution (D_2O); (b) Simulated mass distribution (H_2O); (c) Experimental mass distribution.

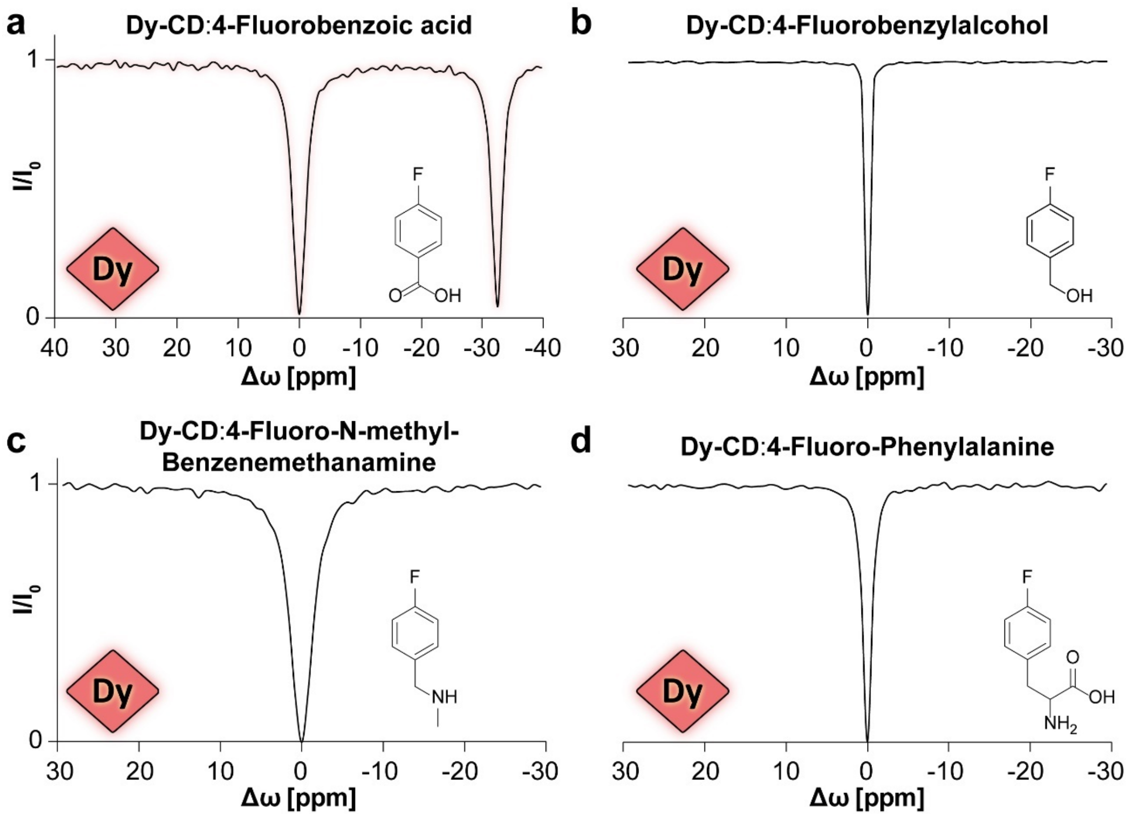

Supplementary Figure 41. z-spectra of paramagnetic Guest Exchange Saturation Transfer (paraGEST) screening experiments for variable guests in Dysprosium-Cyclodextrin (**Dy-CD**, peach diamond) with (a) 4-Fluorobenzoic acid, (b) 4-Fluorobenzylalcohol, (c) 4-Fluoro-N-methyl-Benzenemethaneamine and (d) 4-Fluoro-phenylalanine.

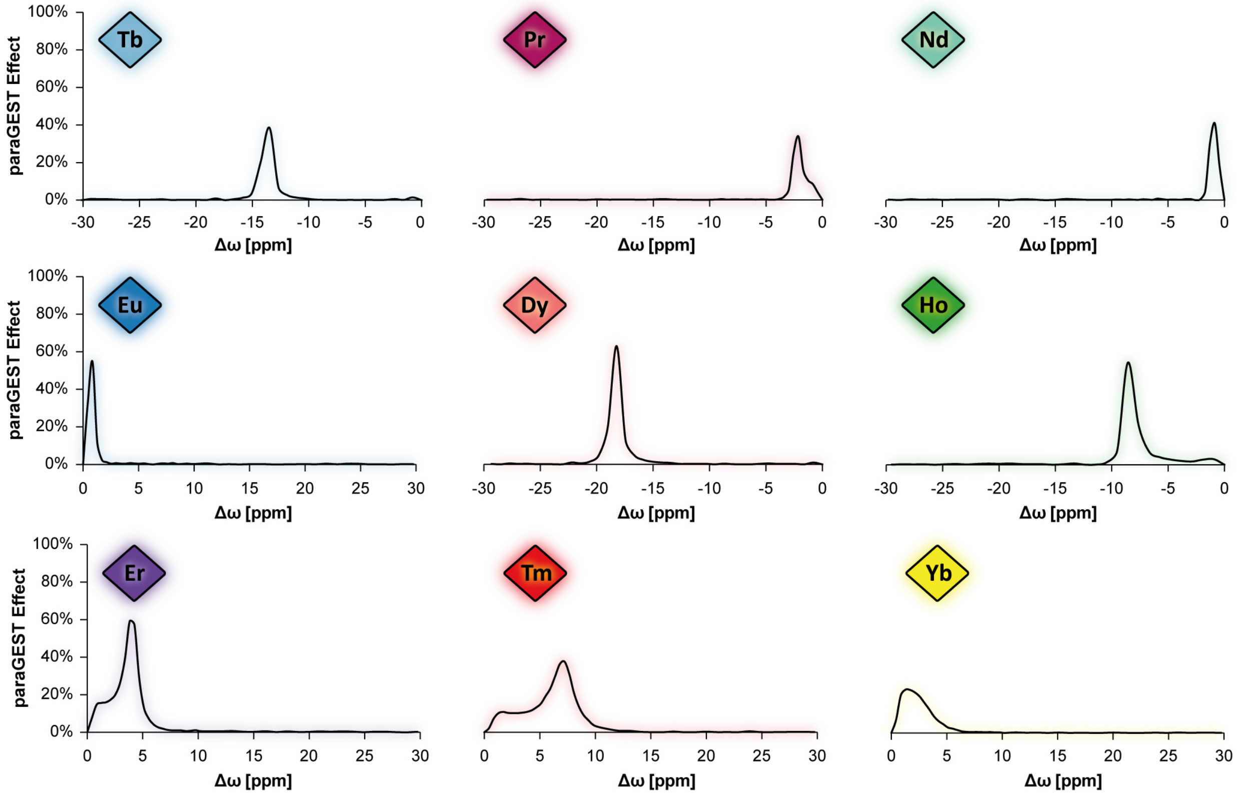

Supplementary Figure 42. Magnetization Transfer Ratio Asymmetry (MTR_{asym}) plots of Lanthanide-Cyclodextrin (**Ln-CD**) hosts (represented by color diamonds) with guest **2** obtained from 1:100 host:guest solutions (Figure 3a).

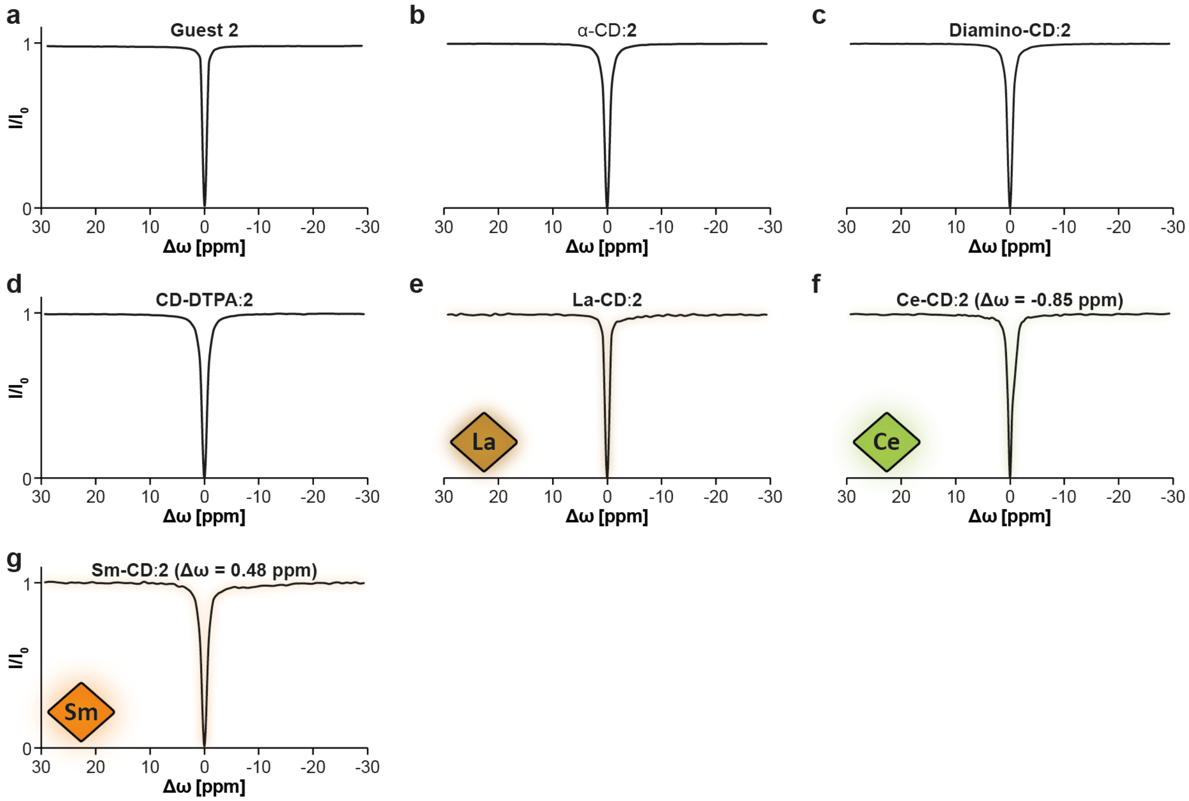

Supplementary Figure 43. z-spectra of control experiments for guest **2**: (a) solution of only guest **2**; guest **2** in the presence of (b) native α-CD; (c) starting material 6^A,6^D-diamino-6^A,6^D-dideoxy-α-cyclodextrin (**Diamino-CD**); (d) product Cyclodextrin diethylenetriaminepentaacetic (**CD-DTPA**); and (e) diamagnetic Lanthanum-Cyclodextrin (**La-CD**, brown diamond); (f, g) z-spectra of paramagnetic Lanthanide-Cyclodextrins (**Ln-CD**s, green and orange diamonds) that exhibit minimal effects for guest **2**, thus were not selected for Magnetic Resonance Imaging (MRI) experiments.

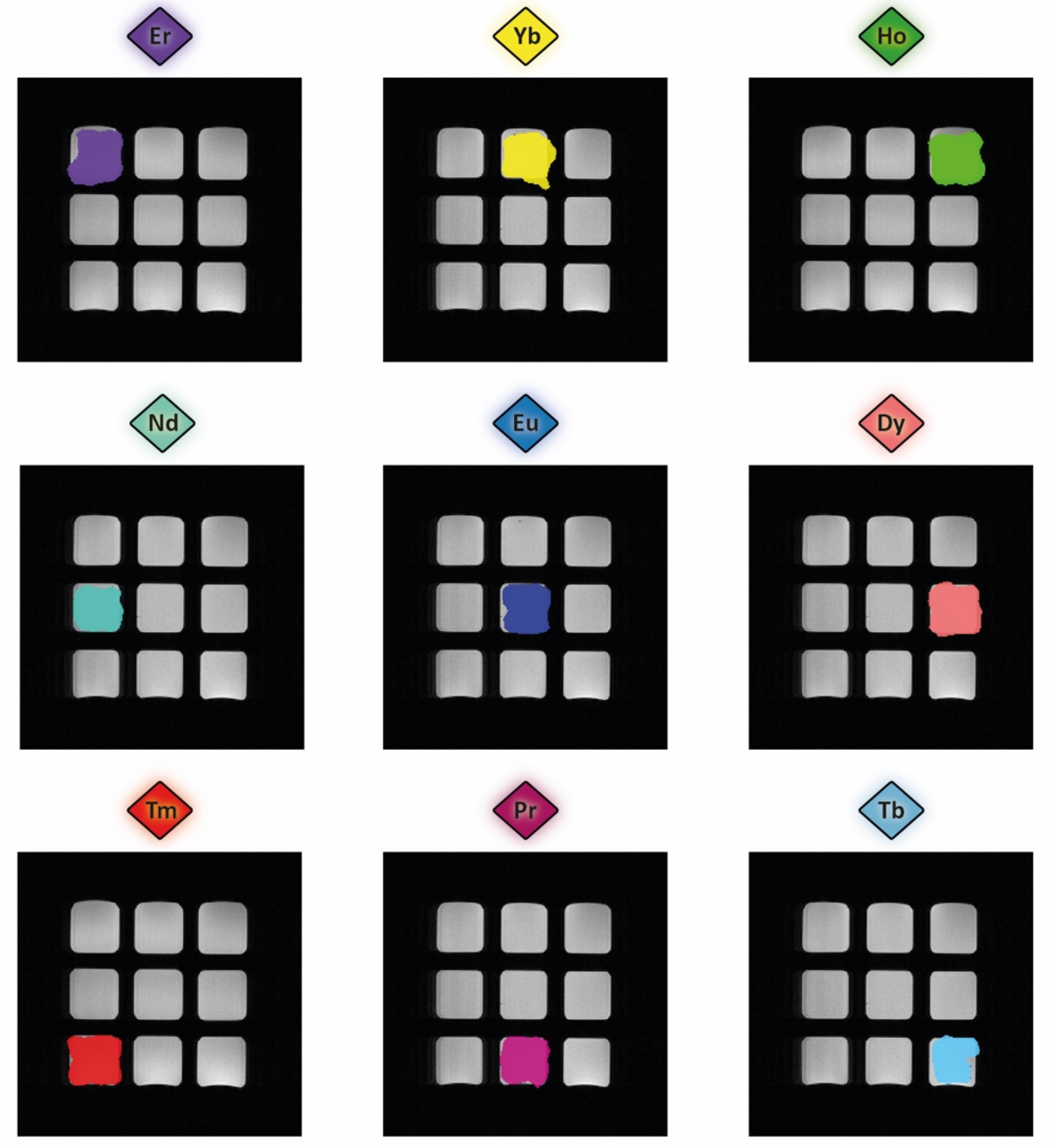

Supplementary Figure 44. Two-dimensional (2D) COlor Display by Exploiting Host-guest Dynamics (CODE-HD) Color-coding. ^{19}Fluorine-paramagnetic Guest Exchange Saturation Transfer (^{19}F-paraGEST) Magnetization Transfer Ratio Asymmetry (MTR_{asym}) maps (color squares) taken at the offsets stated at Supplementary Table 3, overlaid on proton-Magnetic Resonance Imaging (^{1}H-MRI) images (gray squares). Hosts are represented by color diamonds.

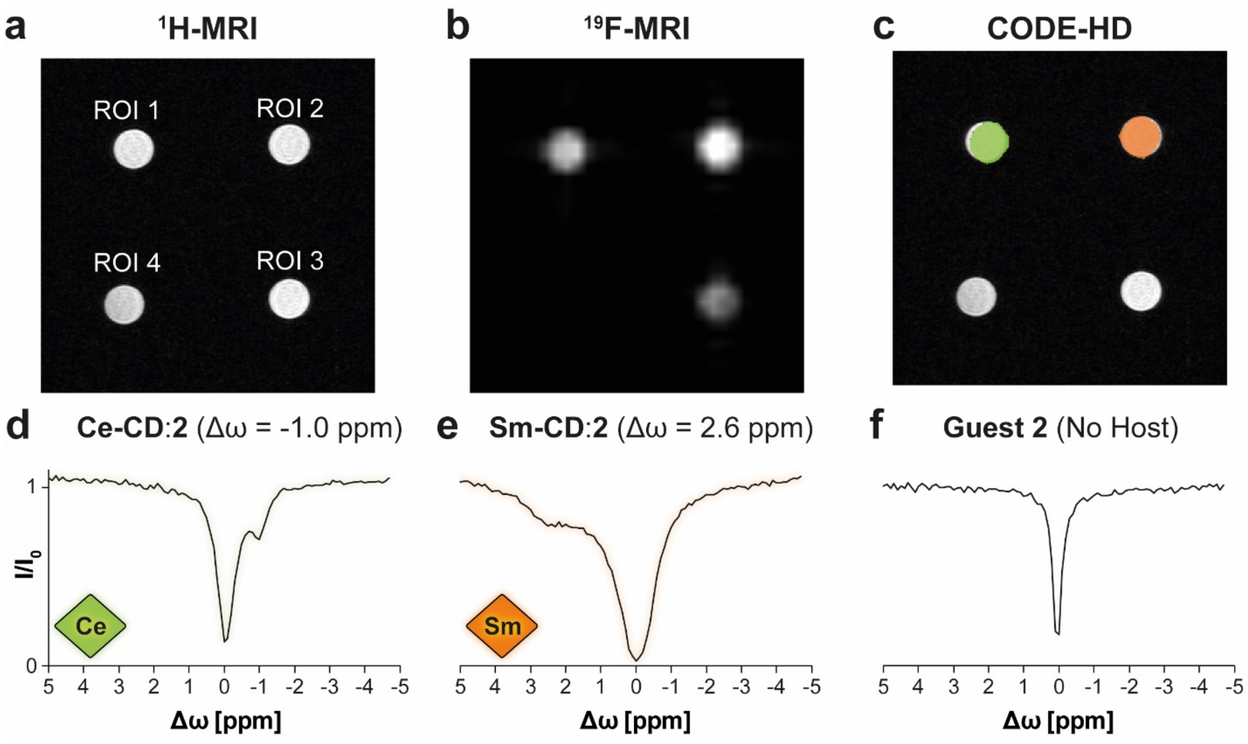

Supplementary Figure 45. Paramagnetic Guest Exchange Saturation Transfer (paraGEST) Magnetic Resonance Imaging (MRI) experiments for Cerium-Cyclodextrin (Ce-CD, green) and Samarium-Cyclodextrin (Sm-CD, orange). (a) Proton-MRI (^{1}H-MRI) map of four 4 cm tubes filled with **Ce-CD**:**2** in a 1:100 ratio (region of interest 1, ROI 1), **Sm-CD**:**2** in a 1:5 ratio (ROI 2), guest **2** without host (ROI 3) and water (ROI 4); (b) ^{19}Fluorine-MRI (^{19}F-MRI) map depicting only tubes with guest **2**; (c) ^{19}F-paraGEST MRI contrast overlaid on ^{1}H-map revealing the tubes filled with **Ce-CD** (green diamond) and **Sm-CD** (orange diamond); z-spectra measured for (d) ROI 1, (e) ROI 2 and (f) ROI 3.

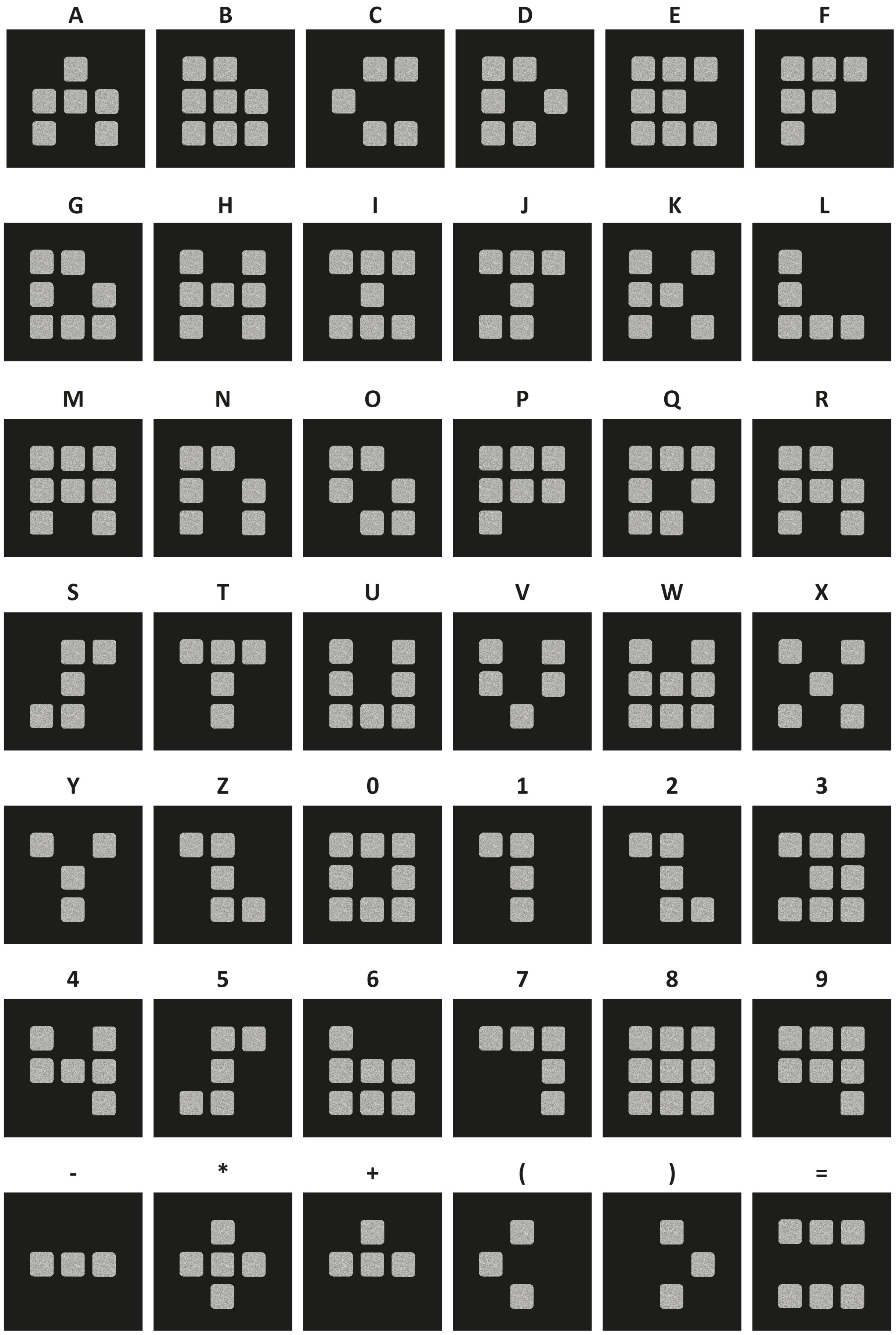

Supplementary Figure 46. 3×3 typeface of Latin alphabet letters, 0-9 digits and additional symbols.

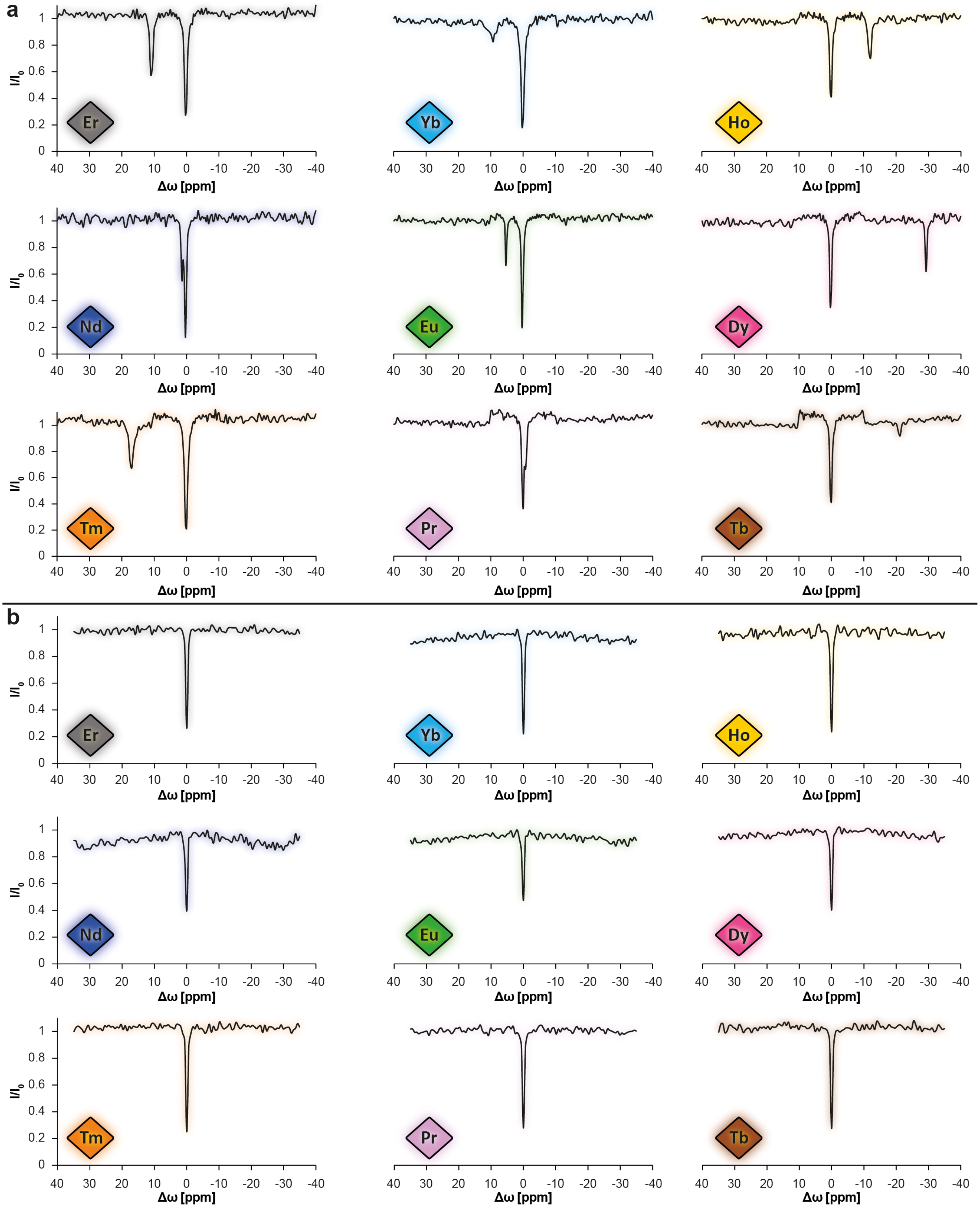

Supplementary Figure 47. (a) z-spectra of Lanthanide-Cyclodextrin (**Ln-CD, color diamonds)**:**3** solutions used for code manipulation (Figure 5b); (b) z-spectra of the same **Ln-CD**:**3** solutions after the addition of guest **5**, used for code deletion (Figure 5c).

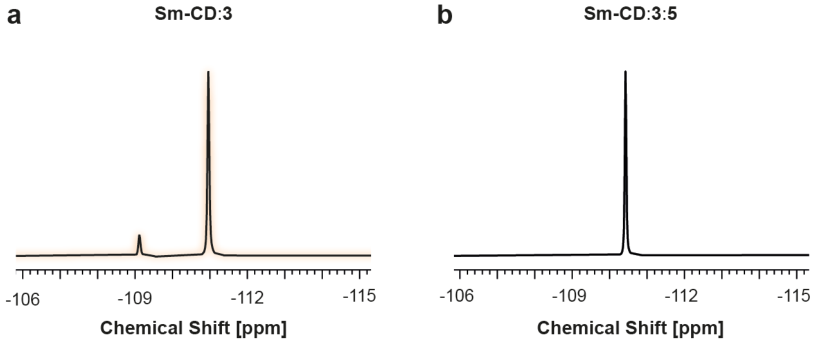

Supplementary Figure 48. ^{19}Fluorine Nuclear Magnetic Resonance (^{19}F-NMR) spectra (470.54 MHz, D_2O, 283K) of Samarium-Cyclodextrin (**Sm-CD**):**3** solution before (a) and after (b) the addition of guest **5**.

SUPPLEMENTARY TABLES

Supplementary Table 1. Cyclodextrin diethylenetriaminepentaacetic acid (CD-DTPA) Proton-Nuclear Magnetic Resonance (^{1}H-NMR) chemical shifts assignment.

Proton Number	Chemical shift [ppm]
H-1	4.94
H-1’, H-1”	4.99, 4.98
H-2, H-2’, H-2”	3.52 - 3.61
H-3, H-3’, H-3”	3.85 - 3.92
H-4	3.52 - 3.59
H-4’, H-4”	3.33 - 3.40
H-5, H-5’, H-5”	3.73 - 3.85
$H-6_a$	3.24 - 3.29
$H-6_b$	4.01
$H-6'_a$	3.58 - 3.62
$H-6'_b$	3.72 - 3.80
$H-6''_a$	3.59 - 3.62
$H-6''_b$	3.70 - 3.73
H-7	4.04 - 4.15
$H-8_{a+b}$	3.09 - 3.22
$H-9_{a+b}$	3.36 - 3.47
H-10	3.84 - 3.92
H-11	3.55 - 3.60

Supplementary Table 2. Cyclodextrin diethylenetriaminepentaacetic acid (CD-DTPA) Carbon-Nuclear Magnetic Resonance (^{13}C-NMR) chemical shifts assignment

Carbon Number	Chemical shift [ppm]
C-1	101.48
C-1’, C-1”	101.60, 101.51
C-2, C-2’, C-2”	71.53, 71.75
C-3, C-3’, C-3”	72.51, 73.44, 73.39
C-4	83.90
C-4’, C-4”	82.05, 81.01
C-5, C-5’, C-5”	72.13, 71.62
C-6	40.82
C-6’	56.78
C-6”	60.68
C-7	54.57
C-8	50.70
C-9	50.60
C-10	60.12
C-11	55.99
C-12, C-13, C-14	170.10, 170.65, 173.05

.

Supplementary Table 3. Artificial colors assigned to COlor Display by Exploiting Host-guest Dynamics (CODE-HD).

Dy	
Δω [ppm]	Color
-18.8	Peach
Tb	
Δω [ppm]	Color
-14.2	Light Blue
Ho	
Δω [ppm]	Color
-8.8	Green
Pr	
Δω [ppm]	Color
-2.3	Purple
Nd	
Δω [ppm]	Color
-1.2	Cyan
Eu	
Δω [ppm]	Color
0.8	Blue
Yb	
Δω [ppm]	Color
2.7	Yellow
Er	
Δω [ppm]	Color
4.2	Dark Purple
Tm	
Δω [ppm]	Color
7.3	Red

Supplementary Table 4. Content of Lanthanide-Cyclodextrin (Ln-CD):2 solutions filled at each well for COlor Display by Exploiting Host-guest Dynamics (CODE-HD) Magnetic Resonance Imaging (MRI) experiments (Figure 3d-f). Total solution volume at each well was set to be 100 µL.

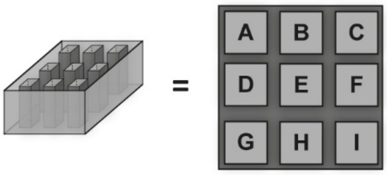

Well	Content
A	**Er-CD**:**2**
B	**Yb-CD**:**2**
C	**Ho-CD**:**2**
D	**Nd-CD**:**2**
E	**Eu-CD**:**2**
F	**Dy-CD**:**2**
G	**Tm-CD**:**2**
H	**Pr-CD**:**2**
I	**Tb-CD**:**2**

Supplementary Table 5. Content of Lanthanide-Cyclodextrin (Ln-CD):2 solutions filled at each well for COlor Display by Exploiting Host-guest Dynamics (CODE-HD) Barcodes encoding experiments (Figure 4a and 4b). Total solution volume at each well was set to be 100 μL.

Barcode I	
Well	Content
A	**Pr-CD**:**2**
B	**Er-CD**:**2**
C	**Dy-CD**:**2**
D	**Er-CD**:**2**
E	**Dy-CD**:**2**
F	**Yb-CD**:**2**
G	**Dy-CD**:**2**
H	**Yb-CD**:**2**
I	**Pr-CD**:**2**
**Barcode II**	
Well	Content
A	**Ho-CD**:**2**
B	**Ho-CD**:**2**
C	**Tb-CD**:**2**
D	**Er-CD**:**2**
E	**Nd-CD**:**2**
F	**Tb-CD**:**2**
G	**Er-CD**:**2**
H	**Tm-CD**:**2**
I	**Tm-CD**:**2**

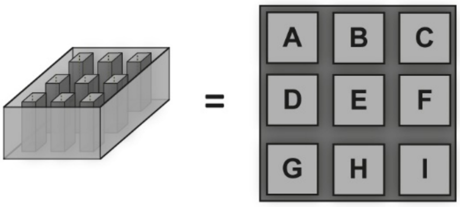

Supplementary Table 6. Content of Lanthanide-Cyclodextrin (Ln-CD):2 solutions filled at each well for COlor Display by Exploiting Host-guest Dynamics (CODE-HD) Words encoding experiments (Figure 4c and 4d). Total solution volume at each well was set to be 100 μL.

CEST	
Well	Content
A	Tm-CD:2, Pr-CD:2
B	Dy-CD:2, Tm-CD:2, Ho-CD:2, Pr-CD:2
C	Dy-CD:2, Tm-CD:2, Ho-CD:2, Pr-CD:2
D	Dy-CD:2, Tm-CD:2
E	Tm-CD:2, Ho-CD:2, Pr-CD:2
F	Guest **2** only
G	Tm-CD:2, Ho-CD:2
H	Dy-CD:2, Tm-CD:2, Ho-CD:2, Pr-CD:2
I	Dy-CD:2, Tm-CD:2
**COLOR**	
Well	Content
A	Yb-CD:2, Er-CD:2, Ho-CD:2
B	Tb-CD:2, Yb-CD:2, Ho-CD:2
C	Tb-CD:2
D	Tb-CD:2, Yb-CD:2, Er-CD:2, Ho-CD:2
E	Ho-CD:2
F	Yb-CD:2, Ho-CD:2
G	Er-CD:2, Ho-CD:2
H	Tb-CD:2, Yb-CD:2, Er-CD:2
I	Tb-CD:2, Yb-CD:2, Er-CD:2, Ho-CD:2

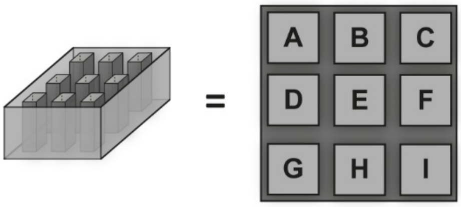

Supplementary Table 7. Content of Lanthanide-Cyclodextrin (Ln-CD):2 solutions filled at each well for COlor Display by Exploiting Host-guest Dynamics (CODE-HD) PIN codes encoding experiments (Figure 4e and 4f). Total solution volume at each well was set to be 100 μL.

1934	
Well	Content
A	**Dy-CD:2**, **Ho-CD:2**, **Tm-CD:2**, **Pr-CD:2**
B	**Dy-CD:2**, **Ho-CD:2**, **Tm-CD:2**
C	**Ho-CD:2**, **Tm-CD:2**, **Pr-CD:2**
D	**Ho-CD:2**, **Pr-CD:2**
E	**Dy-CD:2**, **Ho-CD:2**, **Tm-CD:2**, **Pr-CD:2**
F	**Ho-CD:2**, **Tm-CD:2**, **Pr-CD:2**
G	**Tm-CD:2**
H	**Dy-CD:2**, **Tm-CD:2**
I	**Ho-CD:2**, **Tm-CD:2**, **Pr-CD:2**
*6075	
Well	Content
A	**Pr-CD:2**, **Tm-CD:2**, **Er-CD:2**
B	**Tb-CD:2**, **Tm-CD:2**, **Er-CD:2**, **Yb-CD:2**
C	**Tm-CD:2**, **Er-CD:2**, **Yb-CD:2**
D	**Tb-CD:2**, **Pr-CD:2**, **Tm-CD:2**
E	**Tb-CD:2**, **Pr-CD:2**, **Yb-CD:2**
F	**Tb-CD:2**, **Pr-CD:2**, **Tm-CD:2**, **Er-CD:2**
G	**Pr-CD:2**, **Tm-CD:2**, **Yb-CD:2**
H	**Tb-CD:2**, **Pr-CD:2**, **Tm-CD:2**, **Yb-CD:2**
I	**Pr-CD:2**, **Tm-CD:2**, **Er-CD:2**

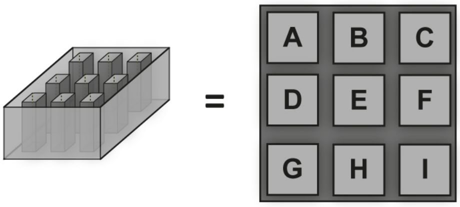

Supplementary Table 8. Artificial colors assigned to Lanthanide-Cyclodextrin (Ln-CD):3 COlor Display by Exploiting Host-guest Dynamics (CODE-HD).

Dy	
Δω [ppm]	Color
-29.2	Hot Pink
Tb	
Δω [ppm]	Color
-21.2	Brown
Ho	
Δω [ppm]	Color
-12.1	Yellow
Pr	
Δω [ppm]	Color
-0.7	Pink Lace
Nd	
Δω [ppm]	Color
1.4	Dark Blue
Eu	
Δω [ppm]	Color
5.4	Green
Yb	
Δω [ppm]	Color
9.6	Light Blue
Er	
Δω [ppm]	Color
11.1	Gray
Tm	
Δω [ppm]	Color
17	Orange

Supplementary Table 9. Content of Lanthanide-Cyclodextrin (Ln-CD):3 solutions filled at each well for code manipulation experiment (Figure 5c). Total solution volume at each well was set to be 100 μL.

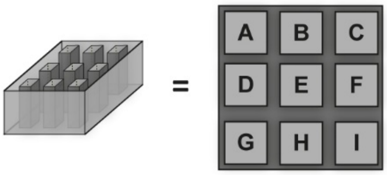

Well	Content
A	**Er-CD:3**
B	**Yb-CD:3**
C	**Ho-CD:3**
D	**Nd-CD:3**
E	**Eu-CD:3**
F	**Dy-CD:3**
G	**Tm-CD:3**
H	**Pr-CD:3**
I	**Tb-CD:3**

Supplementary Table 10. Content of Lanthanide-Cyclodextrin (Ln-CD):2 solutions filled at each well/layer for COlor Display by Exploiting Host-guest Dynamics (CODE-HD) concealed code experiment (Figure 5d).

Top	
Well	Content
A	**Tm-CD:2**
B	**Ho-CD:2**
C	**Tm-CD:2**
D	**Ho-CD:2**
E	**Tm-CD:2**
F	**Ho-CD:2**
G	**Tm-CD:2**
H	**Ho-CD:2**
I	**Tm-CD:2**
Middle	
Well	Content
A	**Er-CD:2**
B	**Yb-CD:2**
C	**Ho-CD:2**
D	**Nd-CD:2**
E	**Eu-CD:2**
F	**Dy-CD:2**
G	**Tm-CD:2**
H	**Pr-CD:2**
I	**Tb-CD:2**
Bottom	
Well	Content
A	**Er-CD:2**
B	**Dy-CD:2**
C	**Er-CD:2**
D	**Dy-CD:2**
E	**Dy-CD:2**
F	**Dy-CD:2**
G	**Er-CD:2**
H	**Dy-CD:2**
I	**Er-CD:2**

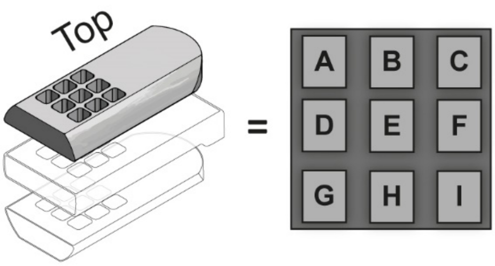

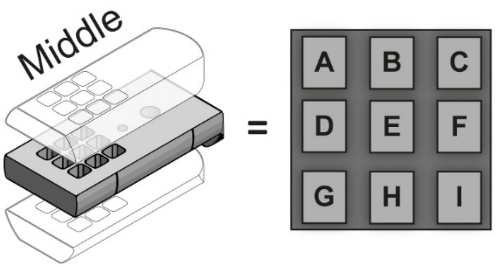

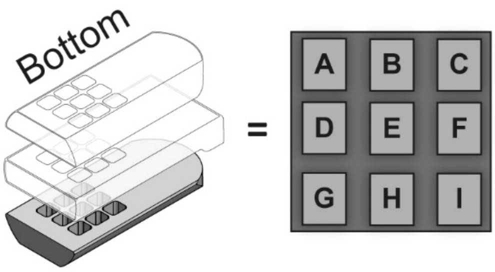

SUPPLEMENTARY REFERENCES

1. Jones CK, *et al.* In vivo three-dimensional whole-brain pulsed steady-state chemical exchange saturation transfer at 7 T. *Magn Reson Med* **67**, 1579-1589 (2012).

2. Liu G, Li Y, Sheth VR, Pagel MD. Imaging in Vivo Extracellular pH with a Single Paramagnetic Chemical Exchange Saturation Transfer Magnetic Resonance Imaging Contrast Agent. *Mol Imaging* **11**, 47-57 (2012).

3. Kim M, Gillen J, Landman BA, Zhou J, van Zijl PC. Water saturation shift referencing (WASSR) for chemical exchange saturation transfer (CEST) experiments. *Magn Reson Med* **61**, 1441-1450 (2009).
